# The burden of disease in Spain: results from the global burden of disease study 2010

**DOI:** 10.1186/s12916-014-0236-9

**Published:** 2014-12-05

**Authors:** Josep Maria Haro, Stefanos Tyrovolas, Noe Garin, Cesar Diaz-Torne, Loreto Carmona, Lidia Sanchez-Riera, Fernando Perez-Ruiz, Christopher JL Murray

**Affiliations:** Parc Sanitari Sant Joan de Déu, Universitat de Barcelona, Fundació Sant Joan de Déu. Dr Antoni Pujades, 42, 08830 Sant Boi de Llobregat, Barcelona, Spain; Instituto de Salud Carlos III, Centro de Investigación Biomédica en Red de Salud Mental CIBERSAM, Dr. Esquerdo 46, 28007 Madrid, Spain; Unitat de Reumatologia. Hospital de la Santa Creu i Sant Pau, Universitat Autònoma de Barcelona, Carrer de Sant Quintí, 89, 08026 Barcelona, Spain; Instituto de Salud Musculoesquelética (InMusc), Calle Hilarión, Eslava 2, 28015, Madrid Spain; Institute of Bone and Joint Research, Royal North Shore Hospital, University of Sydney, Pacific Hwy, St Leonards, New South Wales 2065 Australia; Institut d’Investigació Biomèdica de Bellvitge, Hospital Universitari de Bellvitge, Freixa Llarga s/n, 08907 L’Hospitalet de Llobregat, Barcelona, Spain; Rheumatology Division, Cruces University Hospital, Crystal-induced arthritis investigation team, BioCruces Health Investigation Institute, Plaza de Cruces 12, 48903 Baracaldo, Vizcaya Spain; Institute for Health Metrics and Evaluation, University of Washington, 2301 Fifth Avenue,mSuite 600, Seattle, WA 98121 USA

**Keywords:** Disability-adjusted life years, Global Burden of Diseases, Injuries, and Risk Factors Studies, Spain, Mortality, Years lived with disability, Years of life lost

## Abstract

**Background:**

We herein evaluate the Spanish population’s trends in health burden by comparing results of two Global Burden of Diseases, Injuries, and Risk Factors Studies (the GBD studies) performed 20 years apart.

**Methods:**

Data is part of the GBD study for 1990 and 2010. We present results for mortality, years of life lost (YLLs), years lived with disability, and disability-adjusted life years (DALYs) for the Spanish population. Uncertainty intervals for all measures have been estimated.

**Results:**

Non-communicable diseases accounted for 3,703,400 (95% CI 3,648,270–3,766,720) (91.3%) of 4,057,400 total deaths, in the Spanish population. Cardiovascular and circulatory diseases were the main cause of mortality among non-communicable diseases (34.7% of total deaths), followed by neoplasms (27.1% of total deaths). Neoplasms, cardiovascular and circulatory diseases, and chronic respiratory diseases were the top three leading causes for YLLs. The most important causes of DALYs in 2010 were neoplasms, cardiovascular and circulatory diseases, musculoskeletal disorders, and mental and behavioral disorders.

**Conclusions:**

Mortality and disability in Spain have become even more linked to non-communicable diseases over the last years, following the worldwide trends. Cardiovascular and circulatory diseases, neoplasms, mental and behavioral disorders, and neurological disorders are the leading causes of mortality and disability. Specific focus is needed from health care providers and policy makers to develop health promotion and health education programs directed towards non-communicable disorders.

## Background

The impact of diseases and injures on population health is usually assessed with measures of mortality and non-fatal health outcomes [1]. These estimates are used to signal the most relevant public health problems, allow comparison between different populations and different health conditions, and assess changes over time. The only comprehensive effort to date to estimate summary measures of the global population health, by cause and by world region, is the ongoing Global Burden of Diseases, Injuries, and Risk Factors (GBD) initiative [2,3].

The first GBD study analyzed data from 1990 [4] and was published in 1993. Since then, a number of updates have been published [5]. The Global Burden of Diseases, Injuries, and Risk Factors Study 2010 (GBD 2010) has updated and expanded previous efforts to include 1,160 diseases and injury sequelae from the previous analysis, which included 483 diseases. The most important limitation of previous GBD studies is that results were not estimated with uncertainty [6]. Specifically, uncertainty can come from many sources, including heterogeneity in the empirical data that are available and uncertainty in the indirect estimation models used to make predictions for populations with little or no data. However, this limitation has been solved in the recent analysis of the GBD 2010 study.

The GBD initiative uses disability-adjusted life years (DALYs) and mortality as the summary measurements of the impact of health conditions on population health. The DALY combines the years of life lost (YLLs) due to premature mortality and the years lived with disability (YLDs). The use of DALYs as measure of impact, the inclusion of a large number of communicable and non-communicable conditions, the analysis of the impact of health conditions stratified by gender and age, and the effort to use comparable methodologies across countries and regions make the GBD project an excellent tool to understand the determinants of health and their variability across time and regions. According to previous reported global results, in 1990, 47% of DALYs were attributed to communicable, maternal, neonatal, and nutritional disorders, 43% to non-communicable diseases, and 10% to injuries, while in 2010, this had reversed to 35%, 54%, and 11%, respectively [5]. Besides clear differences among countries with different socioeconomic conditions, heterogeneity is also present when analyzing and comparing countries with more similar socioeconomic conditions. For example, in the United Kingdom (UK), mortality and disability as well as overall health has improved in absolute terms between 1990 and 2010. However, according to Murray et al. [7], the UK performed worse than other EU countries in age-standardized mortality, YLLs, and life expectancy rates. The UK age-standardized DALY rates for chronic obstructive pulmonary disease, disorders of drug use, lower respiratory infections, breast cancers, and preterm birth complications were significantly higher compared to the mean of the EU-15 area countries [7].

Spain is a southern European country with distinct sociodemographic and health characteristics; while in former years large proportions of the population adhered to a Mediterranean diet, dietary habits are changing rapidly with alarming rates of obesity and smoking and alcohol drinking are still highly prevalent in the country despite public health efforts to reduce consumption. Nevertheless, the, until recently, universal coverage of the National Health System also facilitates good health care levels and the implementation of some preventive actions. Until now, to the best of our knowledge, only two studies have tried to assess the burden of disease in Spain. Genova-Maleras et al. [8] estimated the impact of different diseases using DALYs. According to the researchers, the DALYs due to all diseases and injuries were estimated at 5.1 million in Spain. Almost 90% of these were attributed to chronic diseases. Specifically, the leading causes of DALYs were neurological and mental disorders, followed by malignant neoplasms and cardiovascular diseases [8]. However, the aforementioned results were partially limited, since the mortality data came from Spanish registers, while the incidence and severity rates were obtained from estimations of the WHO for a variety of European countries (i.e., WHO European Region Eur-A). A more recent project, but only including data from Valencia, revealed similar results [9], reporting that the number of DALYs gradually increased with age and almost 27% of all DALYs occurred among people over the age of 70 [9].

Given the scarcity of analysis of the data from Spain and the relevance of learning from the experience and comparison with similar and not so similar countries, the aim of the present work was to evaluate the change in burden of disease in Spain, comparing the available data of the GBD over time, between 1990 and 2010.

## Methods

### Overview

The GBD 2010 study estimates the impact of 291 diseases and injuries and 67 risk factors for 187 countries distributed into 21 world regions between 1990 and 2010. For each cause, 1 to 24 sequelae were defined. Sequelae are the clinical outcomes that can be related to specific diseases and injuries such as neuropathy due to diabetes. In total, the study includes 1,160 sequelae. More detailed information about data and analysis for the GBD 2010 have been previously reported [2,5,6,10,11]. For the present analysis, only information regarding Spain will be reported.

### Measurements

We report data on mortality, YLLs due to premature mortality, YLDs, and DALYs. Age-specific mortality rates for Spain were estimated for each sex. As in other developed countries, information about deaths was predominantly driven by data from official vital registration systems [12]. The denominators were based on Spanish census returns and intercensal estimates. Similarly to the other 187 countries of the GBD study, the estimated number of deaths and YLLs was based on 235 defined underlying causes of mortality from the list of 291 diseases and injuries, for 20 age groups and both sexes [2,13]. The YLLs were computed by multiplying the number of deaths in each age group by a reference life expectancy [2]. The YLDs were computed by multiplying the prevalence of a sequelae by its disability weight (DW), used to quantify population’s health losses. This procedure varies slightly compared to previous GBD studies, in which incidence and average duration of the case until remission was used instead of prevalence [14]. A total of 1,160 possible sequelae of diseases and injuries were analyzed. Murray et al. [5] have described the systematic analysis of available data conducted for each sequelae with regard to the prevalence, incidence, remission, and excess mortality. For each age-sex-year group, estimates were made for most sequelae using Bayesian meta-regression methods. DWs were obtained for 220 health states covering the 1,160 sequelae [6]. For each sequelae, DWs were derived based on the scoring of short lay descriptions of the relevant health domains in large population-based studies in several countries (i.e., Peru, USA) and through an open internet survey [15]. Finally, for the estimation of the DALYs, the arithmetic sum of YLLs and YLDs was used.

The YLDs age-standardized rates for each cause, in 1990 and 2010, were calculated. For this procedure the WHO age-standard was used as has been described in former GBD analyses [16]. Spain’s YLDs age-standardized rankings were compared with other European countries. The aforementioned comparison of YLDs age-standardized rates provides an opportunity to compare the YLDs across the European countries in specific periods, controlling for number variations and crude rates due to differences in population age.

In order to differentiate the change in DALYs due to demographic variations from those to health changes or other reasons, two counterfactual increases in total DALYs were calculated based on the 1990 population sex and age distribution and compared with the observed increase from 1990 to 2010: i) the expected increase in total DALYs if total population increase had been as observed but without change in the age/sex structure or in strata-specific DALY rates; ii) the expected increase in total DALYs if the population and its age/sex structure had changed as observed but without change in strata-specific DALY rates (application of 1990 stratum-specific DALY rates to the 2010 population strata sizes). The first estimate, (i), provides the increase attributable to population increase without population aging; (ii) minus (i) gives the increase attributable to population aging and the observed increase from 1990 to 2010 minus (ii) gives the increase attributable to changes in stratum-specific DALY rates.

### Uncertainty levels

Uncertainty levels for mortality rate were estimated using standard simulation methods [2]. Uncertainty for mortality and YLLs reflected uncertainty in the levels of all-cause mortality and uncertainty in the estimation of each mortality cause, in each age group, sex, and year. Uncertainty in the disability weight for each sequelae was propagated into the estimates of YLDs for each disease and injury. For a more accurate estimation of YLDs, the effect of comorbidity was taken into account, as explained in Vos et al. [10]. Specifically, the procedure of microsimulation for each country (explicitly here for Spain), age, sex, and year were used in a large number of simulated individuals. This standard simulation method was repeated 1,000 times to be able to capture uncertainty in the prevalence of all sequelae and disability weights [10].

## Results

From 1990 to 2010, the overall Spanish population increased by almost 15% (from 38,914,907 to 44,558,264 people) (Table 1). A similar increase was observed in males and females, close to 15% and 14%, respectively. The population of older adults in Spain presented the highest increase among all age groups. For example, in octogenarians (aged 80+ years), the increase was almost double. The highest decrease of population was observed in the younger ages (0–20 years old), where the population dropped by 21%.

**Table 1:** **Spanish population (millions) in 1990 and 2010**

	**1990**	**2010**	Δ**%**
Both sexes, all ages	38,914,907	44,558,264	15
0–19 years old	11,078,521	8,781,618	−21
20–39 years old	11,618,044	13,004,297	12
40–59 years old	8,820,927	12,589,546	43
60–79 years old	6,240,651	7,878,252	26
80+ years old	1,156,764	2,304,554	99
Males, all ages	19,057,590	21,964,405	15
Females, all ages	19,857,317	22,593,859	14

Table 2 illustrates the main causes of mortality and YLLs by gender and age group for the Spanish population. As expected, non-communicable diseases were the major cause of mortality, accounting for 3,703,400 (95% CI 3,648,270–3,766,720) (91.3%) of 4,057,400 total deaths. Cardiovascular and circulatory diseases were the main cause of mortality among the non-communicable diseases (34.7% of total deaths), followed by neoplasms (27.1% of total deaths). The third category, injuries, accounted for 4.1% of Spanish deaths [170,040, 95% CI (148,350–187,710)].

**Table 2:** **Main causes of mortality and years of life lost (YLLs) by age groups and by gender, for the Spanish population in 2010**

**Deaths (hundreds)**										**YLLs (hundreds)**		
	**Both sexes, all ages**	**<1 year**	**1–19 years**	**20–39 years**	**40–59 years**	**60–79 years**	**80+ years**	**Males, all ages**	**Females, all ages**	**Both sexes, all ages**	**Males, all ages**	**Females, all ages**
All causes	4,057.4	16.9	14.9	74.9	345.6	1,283.0	2,322.2	2,049.9	2,007.5	54,562.8	32,736.3	21,826.5
**Communicable, maternal, neonatal, and nutritional disorders**	183.96 (157.53–214.88)	10.4	1.3	5.8	14.3	35.6	116.6	89.84 (74.84–110.39)	94.12 (75.54–117)	3,124.9 (2,873.32 – 3,397.56)	1,818.74 (1,629.9 – 2,038.1)	1,306.17 (1,154.6 – 1,471.72)
HIV/AIDS and tuberculosis	16.76 (14.7–19.39)	0.0	0.0	3.6	7.5	3.0	2.8	12.12 (10.21–14.78)	4.63 (3.85–5.63)	555.31 (480.43–649.07)	420.04 (349.4–512.85)	135.27 (115.48–159.21)
Diarrhea, lower respiratory infections, meningitis, and other common infectious diseases	138.1 (113.55–169.75)	0.4	0.7	1.5	5.2	27.8	102.4	64.36 (49.5–85.36)	73.75 (55.79–96.84)	1,432.97 (1,244.82 – 1,680.97)	783.48 (640.77–986.07)	649.49 (534.56–788.48)
Neglected tropical diseases and malaria	1.66 (0.84–3.19)	0.0	0.0	0.0	0.0	0.3	0.5	0.62 (0.31–1.14)	1.04 (0.4–2.45)	49.34 (27.47–89.06)	20.64 (11.35–36.68)	28.7 (11.85–61.85)
Maternal disorders	0.32 (0.22–0.42)	0.0	0.0	0.0	0.0	0.0	0.0	–*	0.32 (0.22–0.42)	16.2 (10.86–21.52)	–*	16.2 (10.86–21.52)
Neonatal disorders	9.8 (8.54–11.03)	9.8	0.0	0.0	0.0	0.0	0.0	5.5 (4.63–6.4)	4.3 (3.4–5.11)	842.8 (734.44–948.51)	473.06 (398.16–550.48)	369.74 (292.36–439.4)
Nutritional deficiencies	9.52 (5.24–13.19)	0.0	0.0	0.0	0.0	1.3	7.8	3.24 (1.82–4.57)	6.29 (2.72–9.74)	82.92 (48.12–106.53)	35.8 (20.2–45.91)	47.12 (22.07–68.11)
Other communicable, maternal, neonatal, and nutritional disorders	7.79 (5.07–9.57)	0.0	0.0	0.1	1.1	3.1	3.1	4 (2.37–5.02)	3.8 (2.32–5.06)	145.36 (100.68–168.33)	85.71 (56.07–102.53)	59.64 (39.63–73.75)
**Non-communicable diseases**	3,703.42 (3,648.27 – 3,766.72)	6.1	7.5	39.6	297.0	1.205.7	2.147.2	1,853.9 (1,818.92 – 1,891.49)	1,849.53 (1,807.93 – 1,895.78)	46,970.31 (46,249.68–48,068.86)	27,624.78 (27,059.44 – 28,507.45)	19,345.53 (18,957.63 – 19,922.49)
Neoplasms	1,101.24 (1,014.78 – 1,168.33)	0.0	3.2	16.5	166.4	513.7	401.3	671.72 (603.52–728.99)	429.52 (386.58–469.72)	18,488.09 (17,216.3 – 19,429.35)	11,636.36 (10,542.8 – 12,486.19)	6851.73 (6324.27–7307.47)
Esophageal cancer	19.45 (14.41–26.46)	0.0	0.0	0.0	4.9	9.9	4.6	16.47 (11.79–23.21)	2.97 (1.89–4.47)	388.59 (286.84–536.01)	349.47 (253.61–493.6)	39.11 (23.43–53.73)
Stomach cancer	64.9 (49.72–93.74)	0.0	0.0	0.7	7.9	29.2	27.1	36.68 (24.69–52.52)	28.22 (18.13–51.95)	986.61 (741.61 – 1,378.18)	613.71 (403.16–859.83)	372.9 (229.02–636.47)
Liver cancer	52.14 (42.39–65.26)	0.0	0.0	0.3	6.8	26.6	18.2	32.93 (25.09–42.88)	19.2 (14.55–28.42)	837.17 (701.49 – 1,036.73)	591.1 (470.26–768.21)	246.07 (197.75–342.93)
Larynx cancer	17.38 (9.03–31.26)	0.0	0.0	0.0	4.8	8.9	3.6	16.56 (8.38–30.24)	0.83 (0.38–1.31)	361.52 (190.52–673.47)	348.38 (180.74–659.95)	13.14 (5.59–22.2)
Trachea, bronchus, and lung cancers	191.92 (123.09–218.32)	0.0	0.0	1.3	39.2	105.4	46.0	163.47 (99.12–188.56)	28.45 (12.43–35.7)	3,641.21 (2,366.65 – 4,115.82)	3,088.96 (1,923.35 – 3,509.43)	552.25 (221.23–698.17)
Breast cancer	65.87 (58.98–73.57)	0.0	0.0	2.0	16.6	24.7	22.5	–*	65.87 (58.98–73.57)	1281.35 (1177.3–1410.43)	–*	1,281.35 (1,177.3 – 1,410.43)
Cervical cancer	9.09 (5.98–13.94)	0.0	0.0	0.4	2.9	3.3	2.4	–*	9.09 (5.98–13.94)	207.1 (130.86–301.52)	–*	207.1 (130.86–301.52)
Uterine cancer	12.5 (5.29–17.15)	0.0	0.0	0.0	1.2	6.3	5.0	–*	12.5 (5.29–17.15)	181.01 (81.49–256.02)	–*	181.01 (81.49–256.02)
Prostate cancer	76.48 (43.85–133.33)	0.0	0.0	0.0	1.3	29.5	45.7	76.48 (43.85–133.33)	–*	818.99 (459.29 – 1,372.44)	818.99 (459.29 – 1,372.44)	–*
Colon and rectum cancers	160.31 (126.24–180.29)	0.0	0.0	1.1	16.9	71.9	70.3	90.85 (66.16–104.65)	69.46 (50.39–82.73)	2,323.71 (1,897.63 – 2,571.55)	1,399.22 (1,060.67 – 1,580.73)	924.49 (704.14 – 1,098.13)
Mouth cancer	13.87 (11.62–16.79)	0.0	0.0	0.1	3.7	6.2	3.6	10.33 (8.48–13.11)	3.54 (2.14–4.85)	282.8 (236.88–352.29)	230.16 (187.58–298.5)	52.65 (31.34–70.41)
Nasopharynx cancer	2.61 (1.64–3.53)	0.0	0.0	0.0	0.8	1.2	0.5	1.96 (1.14–2.91)	0.65 (0.31–1.05)	62.2 (39.43–87.48)	49.32 (29.12–74.66)	12.88 (6.14–19.83)
Cancer of other part of pharynx and oropharynx	8.63 (4.93–11.43)	0.0	0.0	0.0	3.2	4.1	1.2	7.5 (4.12–10.7)	1.13 (0.48–1.83)	206.07 (118.56–292.36)	185.2 (104.09–277.98)	20.87 (8.52–33.12)
Gallbladder and biliary tract cancer	19.93 (13.61–30.72)	0.0	0.0	0.0	1.4	9.3	9.0	7.04 (4.22–11.03)	12.89 (7.14–22.96)	269.16 (187.54–408.35)	107.66 (65.4–163.82)	161.5 (88.78–288.73)
Pancreatic cancer	53.44 (38.17–70.44)	0.0	0.0	0.3	7.6	27.4	18.0	27.66 (17.79–36.7)	25.78 (15.56–39.61)	875.5 (637.54 – 1,113.72)	517.94 (335.01–680.03)	357.56 (215.21–544.31)
Malignant melanoma of skin	9.36 (5.58–13.09)	0.0	0.0	0.5	2.2	3.8	2.7	5.14 (2.28–8.31)	4.22 (2.23–6.97)	198.46 (125.2–285.93)	114.49 (55.15–197.21)	83.97 (46.36–131.3)
Non-melanoma skin cancer	6.79 (4.12–11.36)	0.0	0.0	0.0	0.3	1.6	4.7	3.57 (1.72–6.74)	3.23 (1.42–7.1)	72.93 (44.47–125.29)	46.17 (22.35–90.37)	26.75 (11.84–56.5)
Ovarian cancer	21.15 (13.35–27.17)	0.0	0.0	0.4	5.0	10.1	5.6	–*	21.15 (13.35–27.17)	415.83 (277.13–553.19)	–*	415.83 (277.13–553.19)
Testicular cancer	0.66 (0.32–1.1)	0.0	0.0	0.0	0.0	0.0	0.1	0.66 (0.32–1.1)	–*	21.3 (11.38–31.3)	21.3 (11.38–31.3)	–*
Kidney and other urinary organ cancers	34.18 (24.57–56.41)	0.0	0.0	0.2	5.2	16.8	11.8	21.44 (14.98–41.31)	12.74 (7.01–23.93)	574.91 (420.5–941.19)	385.7 (278.74–736.03)	189.21 (108.55–335.81)
Bladder cancer	49.77 (34.6–60.6)	0.0	0.0	0.0	3.6	22.1	24.1	39.86 (24.7–50.26)	9.91 (7.13–13)	654.29 (461.6–771.51)	549.7 (357.15–673.67)	104.6 (77.76–128.75)
Brain and nervous system cancers	35.98 (19.75–47.79)	0.0	0.9	2.0	8.4	17.2	7.5	19.01 (7.63–26.36)	16.96 (6.63–25.86)	830.9 (476.65 – 1,032.05)	470.77 (201.2–635.62)	360.13 (155.29–483.64)
Thyroid cancer	3.59 (2.59–4.71)	0.0	0.0	0.0	0.3	1.7	1.4	1.2 (0.67–1.73)	2.39 (1.48–3.39)	57.39 (42.19–73.87)	23.49 (12.68–33.42)	33.91 (21.28–47.35)
Hodgkin's disease	2.65 (1.69–3.95)	0.0	0.0	0.3	0.4	1.1	0.6	1.43 (0.72–2.35)	1.22 (0.6–2.06)	70.16 (46.16–106.97)	41.58 (21.53–69.86)	28.59 (14.86–49.59)
Non-Hodgkin lymphoma	27.11 (17.91–33.76)	0.0	0.0	1.0	4.0	12.6	9.3	14.12 (8.57–17.92)	12.98 (6.48–17.22)	487.31 (353.12–563.21)	283.95 (184.07–336.15)	203.35 (113.58–246.19)
Multiple myeloma	17.81 (11.24–25.25)	0.0	0.0	0.0	1.6	9.7	6.4	8.93 (4.6–15.05)	8.88 (4.49–14.08)	267.24 (175.37–381.15)	142.53 (75.4–242.08)	124.71 (64.53–196.93)
Leukemia	39.1 (27.64–48.47)	0.0	0.9	1.5	3.8	16.3	16.6	22.79 (13.64–28.19)	16.31 (8.25–23.37)	672.27 (529–803.1)	406.11 (272.07–491.1)	266.16 (149.02–354.36)
Other neoplasms	84.59 (64.71–111.97)	0.0	0.8	2.2	11.6	37.0	32.9	45.63 (29.95–69.82)	38.96 (25.69–58.6)	1,442.11 (1,134.05 – 1,965.74)	850.47 (589.15 – 1,306.45)	591.64 (403.56–900.02)
Cardiovascular and circulatory diseases	1,411.99 (1,323.93 – 1,541.5)	0.2	0.9	9.8	70.4	374.6	956.1	621.16 (583.59–657.16)	790.83 (714.8–921.4)	1,4937.05 (1,4229.38 – 1,6134.05)	8,344.88 (7,929.4 – 8,713.27)	6,592.17 (6,047.73 – 7,708.66)
Rheumatic heart disease	36.79 (29.03–45.78)	0.0	0.0	0.1	1.3	9.4	25.7	8.77 (5.83–11.22)	28.02 (21.11–36.12)	368.29 (308.32–428.92)	117.17 (78.95–138.38)	251.12 (204.52–302.84)
Ischemic heart disease	626.56 (577.22–692.18)	0.0	0.1	4.9	40.2	178.0	403.3	308.45 (282.37–349.41)	318.11 (282.55–370.95)	7,081 (6,637.38 – 7,802.67)	4,428.89 (4,114.2 – 4,977.74)	2,652.11 (2,409.27 – 3,107.44)
Cerebrovascular disease	429.4 (368.79–561.38)	0.0	0.1	1.9	14.0	103.2	310.0	171.63 (142.42–222.99)	257.77 (213.81–366.07)	4,112.36 (3,640.69 – 5,216.74)	2,003.92 (1,732.92 – 2,516.07)	2,108.44 (1,807.93 – 2,964.23)
Hypertensive heart disease	63.64 (47.68–83.36)	0.0	0.0	0.1	1.6	12.9	48.8	21.25 (15.45–26.68)	42.38 (27.82–62.1)	566.48 (445.62–709.6)	242.98 (183.82–285.61)	323.5 (216.24–460.26)
Cardiomyopathy and myocarditis	50.88 (43.67–60.5)	0.0	0.0	1.1	4.8	16.3	28.4	28.21 (22.58–36.59)	22.67 (18.15–27.22)	697.3 (611.01–826.99)	470.92 (389.76–589.69)	226.37 (192.37–257.76)
Atrial fibrillation and flutter	49.57 (29.32–78.88)	0.0	0.0	0.0	0.3	8.8	40.5	16.36 (8.17–30.95)	33.21 (16.15–60.01)	388.35 (250.38–588.8)	156.64 (84.08–278.6)	231.71 (129.37–389.33)
Aortic aneurysm	22.1 (16.23–29.05)	0.0	0.0	0.1	1.8	9.8	10.2	16.99 (11.53–23.56)	5.11 (3.65–8.83)	308.17 (224.6–403.06)	251.95 (169.97–345.83)	56.22 (45.13–84.66)
Peripheral vascular disease	13.51 (7.89–21.83)	0.0	0.0	0.0	0.1	2.8	10.5	4.27 (1.77–8.98)	9.24 (4.84–16.38)	113.92 (69.2–182.18)	49.4 (20.22–100.5)	64.52 (37.27–112.09)
Endocarditis	4.85 (3.53–5.85)	0.0	0.0	0.0	0.3	1.6	2.6	2.32 (1.68–2.94)	2.53 (1.48–3.34)	68.91 (52.71–80.35)	39.65 (30.25–47.19)	29.26 (17.32–36.84)
Other cardiovascular and circulatory diseases	114.69 (103.64–128.09)	0.0	0.0	1.1	5.2	31.8	76.3	42.9 (38.16–47.72)	71.79 (61.6–84.61)	1,232.26 (1,151.54 – 1,317.41)	583.36 (538.22–631.15)	648.9 (582.6–724.27)
Chronic respiratory diseases	314.29 (290.3–340.56)	0.0	0.0	0.9	7.7	85.1	220.4	184.71 (168.12–201.48)	129.58 (112.04–149.2)	3,042.65 (2,862.02 – 3,232.05)	1,988.91 (1,853.32 – 2,124.11)	1,053.75 (943.48 – 1,173.44)
Chronic obstructive pulmonary disease	186.69 (161.55–210.12)	0.0	0.0	0.1	4.1	54.2	128.0	127.89 (102.9–151.05)	58.8 (51.86–66.3)	1,816.89 (1,581.96 – 2,035.79)	1,350.53 (1,113.46 – 1,566.59)	466.36 (424.85–510.99)
Pneumoconiosis	14.55 (9.49–21.14)	0.0	0.0	0.0	0.3	4.1	10.1	8.41 (4.68–13.84)	6.14 (3.08–10.78)	142.94 (95.3–207.26)	95.76 (55.91–154.49)	47.18 (24.97–78.07)
Asthma	10.82 (8.31–14.66)	0.0	0.0	0.0	0.5	3.0	7.0	2.48 (1.66–5.71)	8.34 (5.6–10.39)	126.37 (103.62–173.86)	39.76 (28.92–83.48)	86.61 (61.65–101.64)
Interstitial lung disease and pulmonary sarcoidosis	19.91 (12.32–29.59)	0.0	0.0	0.0	1.0	8.9	10.0	10.94 (5.81–19.32)	8.98 (4.04–15.82)	246.47 (167.1–353.69)	145.14 (85.09–237.34)	101.33 (52.57–165.72)
Other chronic respiratory diseases	82.32 (60.89–108.12)	0.0	0.0	0.2	1.6	14.9	65.3	34.99 (24.62–53.3)	47.32 (30.81–69.1)	709.98 (577.64–969.25)	357.72 (274.56–541.95)	352.26 (248.4–502.74)
Cirrhosis of the liver	88.53 (77.98–111.17)	0.0	0.0	2.1	21.5	37.6	27.3	54.89 (46.88–74.57)	33.64 (27.92–44.82)	1,735.13 (1,547.01 – 2,184.28)	1,222.34 (1,061.28 – 1,660.84)	512.79 (438.15–732.49)
Cirrhosis of the liver secondary to hepatitis B	3.68 (3.06–4.61)	0.0	0.0	0.0	0.8	1.6	1.2	1.93 (1.5–2.69)	1.74 (1.33–2.27)	66.56 (55.74–85.12)	40.9 (31.63–56.41)	25.66 (19.88–34.09)
Cirrhosis of the liver secondary to hepatitis C	38.7 (32.55–49.19)	0.0	0.0	0.4	7.2	17.5	13.6	18.92 (14.59–26.86)	19.78 (15.03–26.26)	672.7 (569.42–857.44)	389.74 (303.2–550.58)	282.96 (221.68–386.87)
Cirrhosis of the liver secondary to alcohol use	38.53 (32.41–50.61)	0.0	0.0	1.1	10.9	15.9	10.6	29.81 (23.69–41.15)	8.72 (6.93–11.34)	812.93 (675.37 – 1,067.51)	676.87 (540.63–931.27)	136.05 (110.95–182.42)
Other cirrhosis of the liver	7.62 (6.34–9.58)	0.0	0.0	0.4	2.7	2.5	1.9	4.24 (3.29–6)	3.39 (2.63–4.49)	182.95 (152.89–235.21)	114.82 (89.6–162.57)	68.12 (53.84–96.43)
Digestive diseases (except cirrhosis)	129.05 (111.24–145.99)	0.0	0.0	0.9	6.1	35.4	86.5	54.43 (41.68–65.06)	74.62 (62.01–87.51)	1,367.61 (1,200.26 – 1,524.33)	712.46 (566.44–835.42)	655.16 (575.25–735.08)
Peptic ulcer disease	7.94 (6.12–12.78)	0.0	0.0	0.0	0.5	2.3	5.0	3.91 (2.75–6.09)	4.03 (2.78–7.21)	94.36 (78.41–133.23)	56.52 (42.89–76.76)	37.84 (29.6–60.57)
Gastritis and duodenitis	0.7 (0.38–1.34)	0.0	0.0	0.0	0.0	0.0	0.5	0.33 (0.15–0.79)	0.37 (0.17–0.83)	7.71 (4.66–14)	4.37 (2.43–9.64)	3.34 (1.85–6.94)
Appendicitis	2.27 (1.25–3.37)	0.0	0.0	0.0	0.0	0.8	1.2	0.99 (0.39–1.73)	1.28 (0.51–2.11)	29.43 (17.11–40.96)	15.49 (6.22–24.66)	13.94 (5.89–20.06)
Paralytic ileus and intestinal obstruction without hernia	18.65 (11.22–27.18)	0.0	0.0	0.0	0.3	4.2	13.9	6.75 (3.24–10.65)	11.89 (6.27–19.31)	168.81 (105.79–226.43)	73.98 (37.43–107.19)	94.82 (52.72–141.84)
Inguinal or femoral hernia	1.98 (1.78–2.05)	0.0	0.0	0.0	0.0	0.4	1.4	0.87 (0.67–0.91)	1.11 (1.07–1.16)	19.83 (17.64–20.41)	10.61 (8.46–10.99)	9.22 (9–9.53)
Non-infective inflammatory bowel disease	3.44 (2.08–4.92)	0.0	0.0	0.0	0.0	1.0	2.1	1.38 (0.77–2.01)	2.05 (1.08–3.31)	42.73 (27.98–55.05)	21.53 (12.89–28.54)	21.2 (12.37–30.96)
Vascular disorders of intestine	31.9 (17.39–57.23)	0.0	0.0	0.0	0.9	8.9	21.9	14.29 (3.42–38.48)	17.61 (7.83–28.97)	311.86 (164.05–601.7)	170.18 (41.36–457.84)	141.68 (65.87–231.55)
Gall bladder and bile duct disease	17.51 (12.19–24.68)	0.0	0.0	0.0	0.3	3.9	13.0	6.69 (3.94–10.06)	10.82 (6.51–17.59)	162.17 (122.46–210.64)	74.29 (46.15–103.25)	87.88 (57.82–129.7)
Pancreatitis	13.45 (9.97–17.26)	0.0	0.0	0.2	1.5	4.7	7.0	6.36 (4.13–9.22)	7.08 (4.72–10.08)	190.96 (146.04–250.94)	113.47 (77.21–169.87)	77.5 (54.84–111.18)
Other digestive diseases	31.22 (21.1–39.68)	0.0	0.0	0.1	1.6	8.9	20.4	12.85 (8.58–16.52)	18.36 (9.95–26.55)	339.75 (246.38–418.1)	172.02 (118.95–215.82)	167.73 (90.77–234.96)
Neurological disorders	298.8 (200.34–394.65)	0.3	0.7	1.7	6.5	64.8	224.8	109.69 (77.39–153.19)	189.11 (95.13–279.64)	2,744.5 (2,092.35 – 3,423.72)	1,220.72 (953.14 – 1,606.4)	1,523.78 (921.34 – 2,077)
Alzheimer's disease and other dementias	243.35 (144.54–342.54)	0.0	0.0	0.0	1.0	44.2	198.0	81.11 (52.94–126.3)	162.24 (67.03–254.46)	1,877.24 (1,253.43 – 2,491)	729.99 (507.22 – 1,079.75)	1,147.26 (548.76 – 1,699.76)
Parkinson's disease	29.53 (18.66–40.27)	0.0	0.0	0.0	0.1	9.3	19.8	14.86 (7.69–21.9)	14.67 (7.22–22.47)	274.27 (188.02–366.69)	151.03 (87.14–227.06)	123.24 (69.48–176.67)
Epilepsy	4.82 (3.25–6.35)	0.0	0.0	0.4	0.8	1.2	2.0	2.47 (1.6–3.31)	2.36 (1.25–3.71)	108.12 (77.25–127.15)	64.85 (42.33–82.13)	43.27 (27.22–55.03)
Multiple sclerosis	1.95 (1.42–2.48)	0.0	0.0	0.0	0.8	0.7	0.2	0.83 (0.54–1.11)	1.12 (0.7–1.57)	54.54 (39.84–70)	23.21 (15.26–31.31)	31.33 (19.49–44.75)
Other neurological disorders	19.16 (12.14–26.18)	0.3	0.3	0.9	3.5	9.2	4.7	10.43 (5.95–15.79)	8.73 (3.82–14.32)	430.33 (278.35–593.29)	251.65 (149.6–392.18)	178.69 (80.22–282.85)
Mental and behavioral disorders	13.49 (9.72–21.5)	0.0	0.1	3.9	4.2	3.2	2.2	10.37 (7.15–18.63)	3.13 (1.83–4.34)	453.07 (323.04–797.96)	375.8 (253.67–729)	77.26 (48.94–109.57)
Schizophrenia	1.17 (0.61–1.69)	0.0	0.0	0.0	0.0	0.4	0.4	0.61 (0.27–0.9)	0.56 (0.22–0.99)	22.53 (12.28–29.9)	13.51 (6.02–19.24)	9.02 (3.73–13.98)
Alcohol use disorders	4.36 (2.61–8.97)	0.0	0.0	0.2	2.0	1.7	0.4	3.82 (2.05–8.28)	0.55 (0.22–1.13)	124.99 (77.51–260.19)	109.43 (61.15–243.7)	15.57 (6.05–31.99)
Drug use disorders	6.98 (3.87–12.75)	0.0	0.1	3.3	2.0	0.6	0.8	5.52 (2.66–10.82)	1.47 (0.54–2.24)	291.52 (165.61–588.06)	245.59 (124.78–541.19)	45.92 (21.12–72.44)
Eating disorders	0.29 (0.09–0.53)	0.0	0.0	0.0	0.0	0.0	0.2	0.1 (0.02–0.2)	0.19 (0.05–0.39)	4.53 (2.04–6.56)	1.77 (0.6–2.79)	2.76 (1.07–4.43)
Other mental and behavioral disorders	0.69 (0.22–1.23)	0.0	0.0	0.0	0.0	0.0	0.4	0.32 (0.08–0.59)	0.37 (0.08–0.77)	9.5 (3.94–14.28)	5.5 (1.79–8.61)	3.99 (1.36–7.01)
Diabetes, urogenital, blood, and endocrine diseases	280.14 (242.63–353.84)	0.3	0.9	2.2	11.2	79.6	185.6	122.76 (103.65–167.15)	157.38 (128.53–205.29)	3,032.98 (2,645.05 – 4,078.86)	1,571.2 (1,318.85 – 2,311.61)	1,461.78 (1,239.48–1,988.42)
Diabetes mellitus	112.02 (93.98–151.69)	0.0	0.0	0.2	3.9	34.7	73.3	44.69 (36.31–64.25)	67.33 (53.47–95.38)	1,146.26 (995.43 – 1,592.83)	561.77 (479.06–867.39)	584.49 (484.08–889.93)
Acute glomerulonephritis	0.12 (0.06–0.2)	0.0	0.0	0.0	0.0	0.0	<0.1	0.07 (0.03–0.13)	0.05 (0.02–0.1)	2.5 (1.13–3.85)	1.55 (0.61–2.75)	0.95 (0.33–1.55)
Chronic kidney diseases	84.59 (67.95–110.54)	0.0	0.0	0.2	2.4	21.6	60.0	40.03 (32.54–56.18)	44.56 (30.42–67.37)	819.87 (698.77 – 1,098.83)	434.71 (369.26–647.87)	385.15 (289.79–590.98)
Urinary diseases and male infertility	44.35 (26.86–63.32)	0.0	0.0	0.0	1.1	10.8	32.0	21.01 (10.9–33.29)	23.34 (11.35–38.22)	422.38 (270.67–566.13)	222.07 (121.63–335.67)	200.31 (108.39–294.38)
Gynecological diseases	0.35 (0.3–0.39)	0.0	0.0	0.0	0.0	0.0	0.2	–*	0.35 (0.3–0.39)	5.16 (3.99–6.54)	–*	5.16 (3.99–6.54)
Hemoglobinopathies and hemolytic anemia	5.12 (3.77–7.24)	0.0	0.0	0.0	0.0	1.3	3.3	1.89 (1.27–3.33)	3.23 (2.15–4.79)	63.28 (49.79–89.97)	28.65 (21.48–47.37)	34.63 (24.75–52.35)
Other endocrine, nutritional, blood, and immune disorders	33.6 (20.57–84.45)	0.3	0.8	1.3	3.4	11.0	16.8	15.07 (7.43–50.5)	18.52 (9.54–47.65)	573.52 (378.68–1356.21)	322.44 (178.48–991.18)	251.08 (145.49–542.18)
Musculoskeletal disorders	39.06 (25.64–66.48)	0.0	0.0	0.3	1.5	7.9	29.3	13.39 (7.92–29.58)	25.67 (14.36–48.24)	375.55 (268.26–613.79)	143.94 (96.43–294.08)	231.6 (141.74–414.1)
Rheumatoid arthritis	2.89 (1.98–4.19)	0.0	0.0	0.0	0.1	1.5	1.1	0.65 (0.36–0.91)	2.24 (1.42–3.54)	44.56 (31.57–68.08)	11.65 (6.79–15.99)	32.91 (22–57.69)
Other musculoskeletal disorders	36.17 (23.93–62.55)	0.0	0.0	0.2	1.3	6.5	28.2	12.74 (7.32–28.65)	23.43 (13.07–42.38)	330.99 (241.09–564.59)	132.3 (86.48–278.21)	198.69 (129.47–310.74)
Other non-communicable diseases	26.82 (21.12–32.38)	4.9	1.3	1.1	1.7	4.1	13.7	10.76 (8.68–13.33)	16.06 (11.32–21.15)	793.69 (700.07–998.5)	408.18 (349.11–554.42)	385.51 (323.19–522.23)
Congenital anomalies	15.23 (12.89–18.32)	4.2	1.3	1.1	1.5	2.0	5.3	6.84 (5.66–8.92)	8.38 (6.41–10.77)	637.65 (561.04–826.64)	336.37 (283.44–482.44)	301.28 (254.05–437.58)
Skin and subcutaneous diseases	10.88 (6.36–15.66)	0.0	0.0	0.0	0.0	2.2	8.4	3.53 (1.83–5.17)	7.36 (3.26–11.69)	94.99 (60.79–124.7)	37.8 (22.51–50.55)	57.19 (28.45–83.86)
Sudden infant death syndrome	0.71 (0.37–1.3)	0.7	0.0	0.0	0.0	0.0	0.0	0.4 (0.16–0.89)	0.31 (0.12–0.74)	61.05 (31.43–111.36)	34 (14.13–76.52)	27.04 (10–63.55)
**Injuries**	170.04 (148.35–187.71)	0.3	6.1	29.5	34.1	41.7	58.4	106.22 (90.64–120.45)	63.81 (51.39–76.28)	4,467.58 (3,963.64 – 4,986.31)	3,292.76 (2,796.78 – 3,744.15)	1,174.82 (1,034.86 – 1,351.47)
Transport injuries	43.13 (37.17–58.97)	0.1	3.6	12.6	11.0	10.4	5.4	31.86 (25.72–45.25)	11.27 (9.3–16.49)	1,619.03 (1,392.59 – 2,120.51)	1,251.7 (1,009.35 – 1,695.55)	367.33 (311.6–513.85)
Road injury	39.5 (34.03–54.39)	0.0	3.5	11.9	9.9	9.3	4.9	28.91 (23.7–41.92)	10.59 (8.9–16.96)	1,495.72 (1,292.76 – 1,960.84)	1,151.98 (940.51 – 1,613.62)	343.74 (298.04–519.39)
Other transport injury	3.63 (2.55–4.38)	0.0	0.1	0.9	1.1	1.2	0.5	2.95 (1.89–3.64)	0.68 (0.41–0.94)	123.31 (84.2–148.87)	99.72 (65–123.8)	23.59 (13.19–31.95)
Unintentional injuries other than transport injuries	86.75 (66.99–104.08)	0.2	1.5	7.1	10.1	20.6	47.2	44.43 (34.25–53.05)	42.32 (29.03–55.39)	1,528.68 (1,254.66 – 1,728.1)	1,042.66 (815 – 1,229.43)	486.02 (376.59–575.11)
Falls	30.18 (18.17–39.84)	0.0	0.1	1.5	2.9	7.5	18.0	14.31 (7.51–19.64)	15.87 (8.02–23.56)	447.58 (283.13–557.46)	290.71 (160.9–367.9)	156.87 (86.49–210.64)
Drowning	6 (4.88–7.84)	0.0	0.5	1.3	1.6	1.8	0.8	4.75 (3.71–6.73)	1.24 (0.93–1.72)	211.72 (171.89–283.97)	174.93 (136.46–247.02)	36.79 (28.15–50.44)
Fire, heat, and hot substances	3.68 (2.88–5.15)	0.0	0.0	0.1	0.4	0.9	1.9	1.91 (1.41–2.8)	1.78 (1.2–2.94)	70.8 (57.89–97.19)	45.6 (33.42–69.01)	25.2 (18.93–38.16)
Poisoning	4.4 (2.11–6.11)	0.0	0.0	1.6	1.2	0.6	0.8	2.98 (1.07–4.44)	1.42 (0.61–1.99)	158.83 (71.04–225.89)	120.96 (40.98–185.67)	37.88 (18.83–49.55)
Exposure to mechanical forces	3.13 (1.83–4.37)	0.0	0.0	0.7	1.1	0.7	0.4	2.59 (1.37–3.91)	0.54 (0.21–0.84)	110.16 (64.39–170.53)	97.15 (52.91–157.16)	13.01 (6.3–17.41)
Adverse effects of medical treatment	9.25 (5.89–14.86)	0.0	0.0	0.0	0.8	3.0	5.2	3.72 (2.85–4.92)	5.53 (2.38–11.18)	124.7 (92.38–189.32)	61.82 (50.93–75.98)	62.88 (32.01–124.6)
Animal contact	0.23 (0.16–0.33)	0.0	0.0	0.0	0.0	0.0	<0.1	0.17 (0.1–0.25)	0.06 (0.03–0.1)	6.95 (4.67–10.13)	5.57 (3.3–8.48)	1.38 (0.82–2.26)
Unintentional injuries not classified elsewhere	29.87 (20.72–40.43)	0.1	0.1	1.4	2.2	6.0	20.1	14 (10.17–19.39)	15.87 (7.88–25.14)	397.93 (328.14–565.3)	245.92 (194.62–407.48)	152.01 (106.27–205.69)
Self-harm and interpersonal violence	40.16 (29.92–51.78)	0.0	0.6	9.9	12.9	10.7	5.8	29.94 (20.81–40.68)	10.22 (7.11–14.48)	1,319.87 (925.79 – 1,566.26)	998.4 (662.08–1251.41)	321.47 (206.88–412.94)
Self-harm	35.73 (25.84–47.01)	0.0	0.5	8.2	11.5	10.0	5.4	26.91 (17.67–37.57)	8.82 (5.95–13.1)	1,139.19 (743.97 – 1,364.03)	870.51 (539.56 – 1,112.22)	268.69 (162.15–360.64)
Interpersonal violence	4.43 (3.28–6.27)	0.0	0.1	1.7	1.4	0.7	0.4	3.03 (1.99–4.87)	1.4 (1–1.69)	180.68 (131.92–255.73)	127.9 (81.49–197.53)	52.78 (36.94–63.05)

Data are presented as mean values and uncertainty intervals (Lower, Upper).

Mortality’s uncertainty intervals in the six age groups (original age groups are 20) are not included in the table and will be given upon request.

*– not applicable.

Age-specific analysis revealed that non-communicable diseases remained the major cause of mortality except for children below 1 year of age. Cardiovascular- and circulatory-related mortality raised with ageing, from 6% of deaths in individuals younger than 20 years old to almost 41% in those 80 years old and over. Neoplasm-related mortality was almost 22% of deaths in individuals aged up to 40 years, nearly doubled in the group aged 40–79 years and finally dropped to 17% in the group of octogenarians. On the contrary, the main cause of mortality in newborns was communicable diseases, which accounted for 61.5% of all deaths within the group.

**Figure 1:**
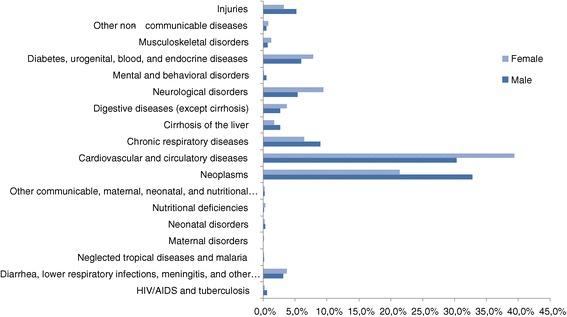
**Spanish deaths in 2010 for males (204,990 total deaths) and females (200,750 total deaths) at all ages.**

In regard to gender differences, the main cause of male mortality was neoplasms (33%), followed by cardiovascular diseases (30.3%), chronic respiratory diseases (9.5%), neurological diseases (5.3%), and injuries (5.2%). On the other hand, the main cause of female mortality were cardiovascular diseases (39.4%) followed by neoplasms (21.4%), neurological disorders (9.4%), and the group formed by diabetes, urogenital, blood, and endocrine diseases (7.8%) (Figure 1).

**Figure 2:**
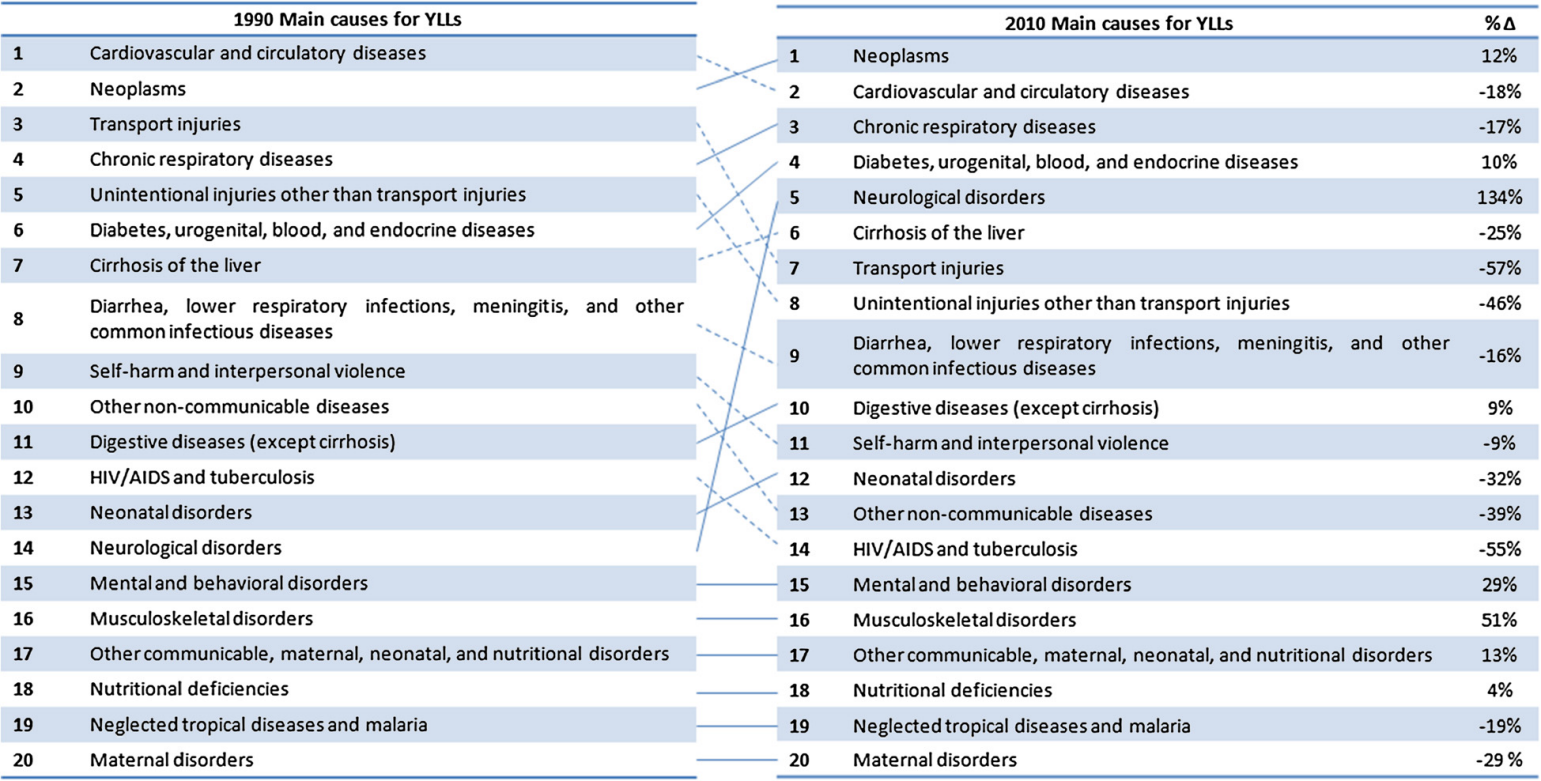
**Spanish years of life lost (YLLs) ranks for the top 20 main causes in 1990 and 2010, and the percentage change between 1990 and 2010.** Solid line for increase or equal position. Continuous line represents an ascending order in rank and the broken line represents a descending order. (Δ%) = % of change in absolute numbers of DALYs.

As shown in Table 2, 86% of total YLLs were due to non-communicable diseases, with injuries and communicable diseases accounting for 8% and 6%, respectively (see specific ranking in Figure 2). In 2010, regardless of gender, the leading specific cause for YLLs was neoplasms followed by cardiovascular and circulatory diseases.

In the top twenty causes of YLLs, the ranking (% of change between the number of YLLs) changed between 1990 and 2010 (Figure 2). Remarkably, due to a large decrease in the number of YLLs, cardiovascular and circulatory diseases dropped from the first cause in 1990 to the second cause of YLLs in 2010 (decreased by 18%). Neoplasms were the leading cause in 2010 (the second in 1990), and increased almost 12% in 2010 compared to 1990. Transport injuries were the seventh cause (third in 1990), and the chronic respiratory diseases were the third cause (fourth in 1990). Unintentional injuries other than transport injuries were the eighth cause (fifth in 1990), and decreased by approximately 46% since 1990. Diabetes, urogenital, blood, and endocrine diseases were the fourth cause (sixth in 1990), increasing by almost 10%. The burden of YLLs in 2010, attributable to cirrhosis of the liver as well as diarrhea, lower respiratory infections, meningitis, and other common infectious diseases, decreased compared with 1990 (seventh to sixth cause and eight to ninth position, respectively). The percentage of YLLs attributable to self-harm and interpersonal violence, neonatal disorders, other non-communicable diseases, and HIV/AIDS and tuberculosis, decreased by almost 9%, 32%, 35%, and 55% between 1990 and 2010, respectively. In contrast, YLLs attributable to neurological disorders increased by 134% and were the fifth leading cause in 2010, while YLLs due to mental and behavioral disorders increased by 29% between 1990 and 2010 (Figure 2).

Table 3 presents all causes of DALYs and YLDs in the Spanish population between 1990 and 2010. In this 20-year period, there was an increase in the overall burden of YLDs of almost 26%. In the broader classification terms (between 1990 and 2010), musculoskeletal disorders, mental and behavioral disorders, and diabetes, urogenital, blood, and endocrine diseases were the three main contributors to the years lived with disability in Spain. Focusing only in 2010, the largest contributors to the burden of YLDs, were the musculoskeletal disorders (26%) and the mental and behavioral disorders (22%). The group of diabetes, urogenital, blood, and endocrine disorders had an important contribution to the burden of disability (10%), followed by the group of other non-communicable disorders (9%), neurological disorders (8%), and other unintentional injuries except for transportation injuries (7%).

**Table 3:** **Comparison of the main causes of disability-adjusted life years (DALYs) and years lived with disability (YLDs), in 1990 and 2010, for the Spanish population**

**YLDs (hundreds)**				**DALYs (hundreds)**		
	**1990**	**2010**	**% Δ**	**1990**	**2010**	**% Δ**
All causes	44,316.8	55,706.6	25.7%	104,615.9	110,269.4	5.4%
**Communicable, maternal, neonatal, and nutritional disorders**	1,914.7 (1,415.3–2,628.9)	1,942.5 (1,377.8–2,767.8)	1%	6,394.8 (5,773.6–7,126)	5,067.4 (4,450.8–5,848.2)	−21%
HIV/AIDS and tuberculosis	285.4 (202.5–384.5)	183 (128.5–248.3)	−36%	1,532.1 (1,356.2–1,720.5)	738.3 (643.6–844.3)	−52%
Tuberculosis	114.8 (71.7–171.8)	84.4 (50.8–129.2)	−26%	340.6 (274–409.3)	175.9 (136.8–230.7)	−48%
HIV/AIDS	170.6 (123.2–223.1)	98.6 (70.8–131.4)	−42%	1,191.6 (1,042–1,360.4)	562.4 (478.8–651.9)	−53%
HIV disease resulting in mycobacterial infection	12.1 (6.7–19.4)	4.7 (2–8.3)	−62%	59.8 (51.5–69.1)	19.6 (16–24.1)	−67%
HIV disease resulting in other specified or unspecified diseases	158.4 (114.2–206.7)	93.9 (67.9–125.4)	−41%	1,131.7 (993.6–1,271.3)	542.8 (469.8–629.4)	−52%
Diarrhea, lower respiratory infections, meningitis, and other common infectious diseases	858.5 (590.7–1249)	925.1 (621.7–1339.5)	8%	2,555.5 (2,206.4–3,004.8)	2,358.1 (1,992.4–2,814.4)	−8%
Diarrheal diseases	306.2 (201.3–440.4)	363.2 (238.6–520.2)	19%	358.6 (252.5–493.1)	428.9 (305.6–585.7)	20%
Typhoid and paratyphoid fevers	0 (0–0.1)	0 (0–0.1)	27%	1.1 (0.1–2)	1 (0.1–2)	−4%
Lower respiratory infections	44.3 (29.7–62)	48.4 (33–66.8)	9%	1391.6 (1227.5–1612.5)	1,275.9 (1,096–1,523.9)	−8%
Upper respiratory infections	219.3 (106.2–390.9)	227.8 (110.9–429.7)	4%	221 (107.8–392.7)	228.4 (111.5–430.3)	3%
Otitis media	211.6 (127.1–339.9)	211.9 (120.7–361)	0.2%	213.6 (127.9–341.7)	213.3 (123–363.3)	0%
Meningitis	52.1 (33.7–77.9)	46.8 (29–71.8)	−10%	261.9 (229.8–309)	153.7 (129.1–183.3)	−41%
Pneumococcal meningitis	10.2 (6.2–15.4)	6.7 (3.8–10.6)	−34%	39.8 (33.2–47.3)	21.8 (17.6–26.7)	−45%
H influenza type B meningitis	7.4 (4.3–11.8)	3.1 (1.6–5.1)	−58%	33.7 (27.2–42.1)	13.6 (10.8–17.3)	−60%
Meningococcal infection	16.6 (10.2–25.7)	14.6 (8.4–23.2)	−12%	88.8 (75.5–106.7)	50.1 (40.9–61.3)	−44%
Other meningitis	17.2 (10.9–26.6)	21.8 (13.3–33.6)	27%	99 (86–116.3)	67.7 (55.6–82.7)	−32%
Encephalitis	5.6 (3.3–8.7)	5.5 (3.2–9)	−1%	28.5 (24.7–32.7)	17.9 (14.7–21.8)	−37%
Diphtheria	0 (0–0)	0 (0–0)	−82%	0.9 (0–7.3)	0.3 (0–2.5)	−66%
Whooping cough	2.4 (1.3–3.8)	0.6 (0.3–1.1)	−74%	47.8 (2.3–221.4)	9.3 (0.6–40.9)	−81%
Tetanus	0 (0–0)	0 (0–0)	−69%	3.4 (0.4–13)	0.7 (0.1–2.6)	−80%
Measles	0.8 (0.3–1.4)	0 (0–0.1)	−99%	4 (2.2–6.4)	1.5 (0.7–3.9)	−62%
Varicella	16.2 (8.2–29.1)	20.8 (10.1–39.2)	29%	23.3 (9.2–63.2)	27.3 (11.6–67.5)	18%
Neglected tropical diseases and malaria	8.5 (2.3–21.8)	15.6 (2.5–51.2)	83%	69.6 (39.7–120.3)	64.9 (35.1–120.1)	−7%
Malaria	0.5 (0–3.9)	0.1 (0–0.8)	−75%	0.5 (0–3.9)	0.1 (0–0.8)	−75%
Leishmaniasis	1.3 (0.6–2.7)	1.4 (0.6–2.9)	6%	10.6 (7.4–14.7)	8.4 (5.8–11.5)	−21%
*Cysticercosis	0 (0–0)	–*	–*	0.6 (0–2.2)	–*	–*
Echinococcosis	5.3 (0.8–18.1)	7 (1–25.6)	32%	15.6 (2.2–47.7)	12.1 (2.1–35.5)	−22%
Dengue	0 (0–0)	0 (0–0)	–*	31.8 (9.6–70.5)	30.9 (12.5–68.8)	−3%
Rabies	0 (0–0)	0 (0–0)	13%	5.6 (2.7–12.4)	2 (1.1–3.9)	−64%
Food-borne trematodiases	0.1 (0–0.4)	0.1 (0–0.3)	−31%	0.1 (0–0.4)	0.1 (0–0.3)	−31%
Other neglected tropical diseases	1.3 (0.1–1.6)	7 (0.2–6.2)	425%	4.8 (2.5–7)	11.3 (3–11.7)	133%
Maternal disorders	14.1 (8.9–21.7)	12.5 (7.5–19.6)	−11%	37 (29–47.5)	28.7 (21.7–37.2)	−22%
Maternal hemorrhage	5.9 (3.4–9.5)	5.3 (3–8.5)	−11%	10.3 (7.4–14)	8.5 (6–11.9)	−17%
Maternal sepsis	0.3 (0–1)	0.3 (0–1)	−9%	1.3 (0.8–2.1)	0.9 (0.5–1.6)	−33%
Hypertensive disorders of pregnancy	0.6 (0.3–1.1)	0.7 (0.3–1.2)	9%	3.9 (3–5.1)	2.9 (2.1–3.8)	−26%
Obstructed labor	1.1 (0–3.4)	1.1 (0–3.4)	−2%	2 (0.9–4.3)	1.8 (0.7–4.2)	−12%
Abortion	2 (1.1–3.4)	1.4 (0.7–2.5)	−29%	5.2 (3.9–6.8)	3.4 (2.4–4.6)	−35%
Other maternal disorders	4.1 (2.6–6.2)	3.8 (2.3–5.8)	−8%	14.2 (11.1–18.1)	11.3 (8.4–14.4)	−21%
Neonatal disorders	224.6 (164.9–300.8)	218.4 (162.3–282.8)	−3%	1,468.3 (1,242.3–1,668.6)	1,061.2 (934.7–1,183)	−28%
Preterm birth complications	96.4 (66.9–136.9)	109.4 (75.9–149.6)	13%	788.4 (627.9–938.5)	533 (433–665.9)	−32%
Neonatal encephalopathy (birth asphyxia and birth trauma)	126.6 (89.5–172.7)	107 (77.3–143.6)	−15%	425.4 (336.5–527.3)	297.5 (235.6–375)	−30%
Sepsis and other infectious disorders of the newborn baby	0.1 (0.1–0.3)	0.3 (0.2–0.6)	140%	110.1 (60.9–192.3)	111.2 (60–197.5)	1%
Other neonatal disorders	1.4 (1–2)	1.7 (1.2–2.3)	16%	144.3 (91.2–262.3)	119.5 (67.1–179.2)	−17%
Nutritional deficiencies	444.6 (269.2–712.5)	507.8 (314.2–801.1)	14%	524.2 (347–797.2)	590.7 (394.4–885)	13%
Protein-energy malnutrition	14.3 (9.3–21.2)	22.7 (14.8–31.8)	59%	46.2 (38.5–57.6)	75 (52.4–93.8)	62%
Iodine deficiency	427.1 (251.8–694)	480.5 (285–763.7)	13%	428.8 (253.7–695.7)	482.4 (286.9–765.8)	13%
Iron-deficiency anemia	1.1 (0.5–2)	3 (1–3.2)	171%	41.8 (35.4–54.5)	27.9 (16.9–33.7)	−33%
Other nutritional deficiencies	2.2 (1.8–2.8)	1.5 (0.9–2)	−29%	7.5 (6.3–9.8)	5.3 (3.2–6.9)	−29%
Other communicable, maternal, neonatal, and nutritional disorders	79 (38.7–149.3)	80.1 (35.6–168.3)	1%	208 (161.6–281.2)	225.5 (162.4–323.7)	8%
Sexually transmitted diseases excluding HIV	64.3 (29.6–128.4)	56.9 (26–114)	−11%	91.6 (56.3–157.8)	66.6 (35.2–123.7)	−27%
Syphilis	3.9 (0.2–8.7)	4.8 (0.3–10.7)	21%	25.4 (15.7–39.3)	10.9 (5–18)	−57%
Sexually transmitted chlamydial diseases	25.3 (8.7–60)	22.4 (7.6–53.6)	−12%	26.7 (10.1–61.5)	23.2 (8.3–54.3)	−13%
Gonococcal infection	13.7 (5.2–30.2)	14.5 (5–32.7)	6%	14.8 (6.1–31.3)	15.1 (5.6–33.5)	2%
Trichomoniasis	13.3 (0.1–41.8)	9.2 (0.1–29.1)	−31%	13.3 (0.1–41.8)	9.2 (0.1–29.1)	−31%
Other sexually transmitted diseases	8 (2.9–18.1)	6 (2.5–12)	−25%	11.5 (6.2–21.5)	8.2 (4.5–14.2)	−29%
Hepatitis	12.2 (6–21.9)	12.9 (6.2–22.8)	6%	67.3 (57.9–79)	66 (55.8–77.8)	−2%
Acute hepatitis A	8.3 (3.9–14.7)	7.5 (3.7–13)	−9%	17 (11.2–24.8)	11 (6.5–17.6)	−35%
Acute hepatitis B	2.8 (0.2–9.2)	3.4 (0.2–10.6)	21%	42.8 (26.3–55.3)	24 (12.6–43.4)	−44%
Acute hepatitis C	1 (0.2–2.1)	2 (0.4–4.1)	87%	7.6 (2.8–15.8)	31 (10.5–53.4)	308%
Leprosy	0.1 (0–0.1)	0 (0–0)	−87%	0.1 (0–0.1)	0 (0–0)	−87%
Other infectious diseases	2.5 (0–0.3)	10.3 (0–0.4)	314%	49 (37.6–81.3)	92.8 (39.3–114.7)	89%
**Non-communicable diseases**	38,528.9 (32,278.2–45,503.6)	48,269.1 (40,257.6–56,616.6)	25%	86,260 (79,823.1–93,301.2)	95,239.4 (87,522.2–10,3475.6)	10%
Neoplasms	474.2 (349.1–630.6)	816.4 (597.6–1074.5)	72%	16,917.3 (16,102.7–17,858.3)	19,304.5 (18,003–20,302.6)	14%
Esophageal cancer	3.2 (1.6–5.3)	3.6 (1.8–6.2)	10%	413.9 (310.3–535.2)	392.1 (290.6–538.8)	−5%
Stomach cancer	24.3 (15.3–35.6)	21.2 (12.9–33)	−13%	1,443.2 (1,131–1,931.8)	1,007.9 (765–1,402.6)	−30%
Liver cancer	6.5 (3.9–9.8)	9.6 (5.8–14.8)	47%	720 (581.5–818.5)	846.8 (712.5–1,044.6)	18%
Liver cancer secondary to hepatitis B	0.9 (0.3–1.8)	1.3 (0.4–2.4)	38%	100.9 (80.1–116.3)	119.2 (97.8–152.4)	18%
Liver cancer secondary to hepatitis C	2.8 (1.5–4.7)	3.9 (2–6.6)	40%	287.9 (236.3–332.2)	331.9 (277.3–427.1)	15%
Liver cancer secondary to alcohol use	2.2 (1.1–3.7)	3.6 (1.9–5.9)	64%	253 (201.7–291.3)	301 (253–381)	19%
Other liver cancer	0.6 (0.1–1.4)	0.8 (0.1–1.6)	26%	78.2 (62.9–90.4)	94.7 (74.6–116.3)	21%
Larynx cancer	14 (7.3–24.9)	12.3 (6.5–23.3)	−12%	498.7 (252.2–859.6)	373.9 (202.3–685.2)	−25%
Trachea, bronchus, and lung cancers	34.1 (22.3–49.5)	45.5 (25.9–64.2)	34%	3,138.8 (2,541.8–3,982.3)	3,686.7 (2,408.1–4,161.1)	17%
Breast cancer	122.7 (80.8–187.9)	188.1 (120.8–277.2)	53%	1,496.8 (1,400.8–1,611.5)	1,469.4 (1,340.6–1,619.2)	−2%
Cervical cancer	5.4 (2.9–8.7)	5.2 (2.7–8.6)	−4%	226.8 (143.3–321.6)	212.3 (135.6–306.1)	−6%
Uterine cancer	10.9 (5.7–20.1)	13.6 (5.7–23.1)	24%	174.1 (100.8–316.1)	194.6 (93.5–271.9)	12%
Prostate cancer	40.2 (27–60)	120.3 (78.9–185.9)	199%	609 (373.5–941.4)	939.3 (572.7–1,489.8)	54%
Colon and rectum cancers	63.1 (44.8–85.4)	128.7 (90.3–171.7)	104%	1,675.7 (1,476.6–1,895.5)	2,452.4 (2,019.5–2,711)	46%
Mouth cancer	12.4 (8.2–17.8)	13.8 (8.9–20.3)	12%	307.3 (257.8–337.4)	296.6 (250.1–367.2)	−3%
Nasopharynx cancer	0.9 (0.3–1.9)	1 (0.3–2)	5%	70.2 (40.1–94.4)	63.2 (40.4–88.2)	−10%
Cancer of other part of pharynx and oropharynx	4.1 (2–7)	5.8 (2.8–9.4)	42%	172.5 (100.3–251.3)	211.9 (125.8–299.5)	23%
Gallbladder and biliary tract cancer	3.6 (1.9–6.1)	5.1 (2.5–9)	41%	253.9 (169.6–370.5)	274.3 (193–414.1)	8%
Pancreatic cancer	3.9 (2–6.5)	6.3 (3.4–10.4)	63%	627.8 (480.8–812.9)	881.8 (646.5–1120.7)	40%
Malignant melanoma of skin	4.2 (2.3–7.9)	8.6 (4.7–13.7)	103%	140.1 (94.4–223.2)	207.1 (133–294.9)	48%
Non-melanoma skin cancer	15.6 (10.7–21.9)	38 (25.9–52.8)	143%	89.4 (55.1–125.3)	110.9 (80.5–165.1)	24%
Ovarian cancer	6.2 (3.6–9.8)	9.4 (5.2–14.5)	52%	336.7 (252.6–453.1)	425.2 (287.4–561.8)	26%
Testicular cancer	0.8 (0.2–1.6)	1.4 (0.5–2.9)	86%	22.9 (13.7–34.8)	22.7 (12.9–32.9)	−1%
Kidney and other urinary organ cancers	5.6 (3.1–8.6)	17.7 (10.2–30.6)	218%	255.5 (178.9–327.2)	592.6 (435.2–958.7)	132%
Bladder cancer	31.7 (21.7–43.9)	49.8 (32.2–69.6)	57%	617 (491.5–789.2)	704.1 (508.2–824.7)	14%
Brain and nervous system cancers	11.5 (6.6–17.9)	17.4 (8.7–27.2)	51%	716.6 (488.1–1,011.1)	848.3 (492.4–1,050.7)	18%
Thyroid cancer	2.5 (1.3–4.4)	4.1 (2.2–6.8)	63%	51.9 (40.5–66.8)	61.5 (45.7–78.9)	18%
Hodgkin’s disease	2.4 (1–4.2)	2.1 (0.9–3.9)	−11%	112.8 (73.1–166.8)	72.3 (47.9–109.7)	−36%
Non-Hodgkin’s lymphoma	9.4 (6–13.8)	16.1 (10–23.9)	72%	417.3 (366.4–493.7)	503.5 (364.3–578.3)	21%
Multiple myeloma	5.4 (2.8–9)	8.3 (4.3–13.5)	55%	212.7 (146.8–305.2)	275.6 (182.9–388.8)	30%
Leukemia	10.4 (6.7–15.4)	16.1 (10–23.5)	55%	705.9 (575.5–880)	688.4 (544.1–817)	−2%
Other neoplasms	19.2 (12.4–28.5)	47.2 (30–69.8)	145%	1,409.8 (1,108.1–1,845.1)	1,489.3 (1,190.5–2,016.9)	6%
Cardiovascular and circulatory diseases	1,911.6 (1,475.9–2,423)	2,864.9 (2,268.9–3,499.4)	50%	20,076.7 (18,998.5–20,904.2)	17,802 (16,834.2–19,181.8)	−11%
Rheumatic heart disease	125.4 (77.4–198.2)	139.1 (93.2–207.5)	11%	823.4 (745.2–933.8)	507.3 (429.5–599)	−38%
Ischemic heart disease	737.6 (477.9–1108)	974.8 (636–1420.7)	32%	9,202.7 (8,705.1–9,952.4)	8,055.8 (7,462.7–8,876.3)	−12%
Cerebrovascular disease	337.1 (278.1–393.5)	640.5 (529.3–755.2)	90%	6,437.3 (5,648.1–6,783.7)	4,752.8 (4,252.9–5,829.1)	−26%
Ischemic stroke	271.4 (225–318.7)	515.9 (426.9–609.4)	90%	4,130.9 (3,546.5–4,389.3)	3,011 (2,635.2–4,183.6)	−27%
Hemorrhagic and other non-ischemic stroke	65.6 (54.3–77.7)	124.6 (102.1–147.8)	90%	2,306.5 (2,058.8–2,601.6)	1,741.8 (1,502.3–1,936)	−24%
Hypertensive heart disease	28.3 (17.3–42.8)	46.5 (28.8–71.4)	65%	531.9 (432.3–671.4)	613 (490.4–764.1)	15%
Cardiomyopathy and myocarditis	17.5 (10.3–27.4)	26.6 (16.3–40.9)	52%	537.7 (496.6–569.2)	723.9 (634–857.1)	35%
Atrial fibrillation and flutter	278.3 (186.1–398)	460.9 (306.5–658)	66%	363.2 (269.6–484.1)	849.2 (631.8–1127.7)	134%
Aortic aneurysm	0 (0–0)	0 (0–0)	–*	261.2 (199.7–339.1)	308.2 (224.6–403.1)	18%
Peripheral vascular disease	39.5 (20.1–70.8)	60.7 (30.8–109.7)	54%	83.5 (54.6–124.9)	174.6 (116.3–253.8)	109%
Endocarditis	4.2 (2.4–6.8)	7.4 (4.5–11.6)	75%	74.6 (64–86.9)	76.3 (59.9–88)	2%
Other cardiovascular and circulatory diseases	343.8 (182.1–561.1)	508.7 (275.7–807.5)	48%	1,761.1 (1,592.8–1,992.2)	1,740.9 (1,496.8–2,046.5)	−1%
Chronic respiratory diseases	1,935.1 (1,247–2,764.6)	22,17.7 (14,39.4–31,23.6)	15%	5,623.2 (4,907.3–6,436.1)	5,260.3 (4,434.9–6,184.9)	−6%
Chronic obstructive pulmonary disease	860.7 (5,23.1–1,276.8)	10,99.1 (683–15,94.7)	28%	3,030.1 (2,586.4–3,515.9)	2,916 (2,423.7–3,483.1)	−4%
Pneumoconiosis	8.3 (5.3–12)	15.9 (10–23.7)	92%	195.7 (134.5–283)	158.8 (112.5–223.2)	−19%
Asthma	749.4 (411.4–1,203.2)	791.4 (443–1,253.1)	6%	969.4 (632.4–1423.6)	917.8 (564.5–1377.3)	−5%
Interstitial lung disease and pulmonary sarcoidosis	8.9 (5.9–13.3)	16.1 (10.4–24.3)	81%	158.8 (118.1–251.5)	262.6 (181.8–373)	65%
Other chronic respiratory diseases	307.9 (187–458.9)	295.2 (183.7–431.1)	−4%	1,269.2 (988–1,483.8)	1,005.2 (819.7–1,264)	−21%
Cirrhosis of the liver	34.4 (21.9–49.6)	37.6 (24.3–54)	9%	2,358.1 (1,931.4–2,609.8)	1,772.7 (1585.9–2,231.2)	−25%
Cirrhosis of the liver secondary to hepatitis B	1.3 (0.5–2.5)	1.3 (0.5–2.6)	4%	89.4 (70.2–105.5)	67.9 (57–86.1)	−24%
Cirrhosis of the liver secondary to hepatitis C	13.2 (7.4–21.5)	14.4 (7.7–23.3)	9%	895 (711.6–1049.2)	687.1 (582–870.4)	−23%
Cirrhosis of the liver secondary to alcohol use	15.8 (8.9–25)	17.5 (9.9–28.4)	11%	1,105.7 (847.2–1295.5)	830.5 (694.9–1,084.7)	−25%
Other cirrhosis of the liver	4.1 (2–6.9)	4.3 (2.1–7.6)	4%	268 (211.1–313.4)	187.2 (156.9–238.2)	−30%
Digestive diseases (except cirrhosis)	423.2 (248.1–730.7)	492.4 (313.7–755.8)	16%	1,683 (1,452.6–2,012.8)	1,860 (1,610.1–2,164.5)	11%
Peptic ulcer disease	34 (17.9–64.8)	21.8 (11–46.2)	−36%	251.8 (202.3–293.9)	116.2 (95–164.7)	−54%
Gastritis and duodenitis	29.9 (19.3–44.6)	38.8 (25.8–56.3)	30%	46.9 (33.4–62.6)	46.5 (32.1–65)	−1%
Appendicitis	7.8 (2.8–16.7)	8.3 (3–17.8)	6%	43.7 (30.9–65.7)	37.7 (22.8–52.5)	−14%
Paralytic ileus and intestinal obstruction without hernia	0.6 (0–2.7)	0.6 (0–2.6)	3%	123.3 (96.4–175.4)	169.4 (106.6–226.9)	37%
Inguinal or femoral hernia	28.3 (6.6–84)	28.9 (5.7–81.8)	2%	55.8 (33.9–110.3)	48.7 (25.4–101.4)	−13%
Non-infective inflammatory bowel disease	205.8 (88.9–434.4)	214.3 (109.5–390.4)	4%	243.3 (127.2–473.9)	257 (151.9–435.7)	6%
Vascular disorders of intestine	1 (0.2–2.4)	1.6 (0.3–3.9)	70%	232.9 (117.1–468.5)	313.5 (166–604)	35%
Gall bladder and bile duct disease	36.4 (24.5–51.6)	45.6 (30.7–64.3)	25%	189.2 (161.3–229.6)	207.8 (164.8–260.5)	10%
Pancreatitis	16.3 (5.8–38.3)	37.2 (12.8–83.9)	128%	224.4 (164.8–275.8)	228.1 (177–303.9)	2%
Other digestive diseases	63.2 (37–108.4)	95.3 (60.6–146.9)	51%	271.7 (228–356.4)	435.1 (337.6–529.1)	60%
Neurological disorders	3,407 (2,631.9–4,321.9)	4,627.9 (3,616.3–5,804.5)	36%	4,581 (3,789.1–5,481.9)	7,372.4 (6,127.5–8,666.4)	61%
Alzheimer's disease and other dementias	858.3 (612.7–1,136)	1,640.7 (1,185–2,196.8)	91%	1,433.9 (1,138.4–1,754.3)	3,518 (,2707.8–4,334.5)	145%
Parkinson's disease	71.8 (46–114.3)	115.7 (72.9–189)	61%	230.4 (180.8–300.8)	390 (290.8–496.1)	69%
Epilepsy	292 (215.3–381.1)	324 (242.9–425.2)	11%	399.4 (320.1–491.9)	432.1 (343–538.1)	8%
Multiple sclerosis	37.7 (26.5–51)	54.5 (37.3–74.1)	45%	82.1 (67.6–98.9)	109 (86.3–133.8)	33%
Migraine	1,893.5 (1,198.1–2,731.1)	2,146 (1,339.7–3,101.9)	13%	1893.5 (1198.1–2731.1)	2146 (1339.7–3101.9)	13%
Tension-type headache	117.1 (65.8–188)	144.1 (82.7–233.9)	23%	117.1 (65.8–188)	144.1 (82.7–233.9)	23%
Other neurological disorders	136.7 (102.7–176.2)	202.8 (153.7–261.7)	48%	424.5 (336.1–567.1)	633.1 (480.2–802.4)	49%
Mental and behavioral disorders	10,177.2 (8,247.3–12,325.7)	12,464.1 (10,170.8–15,032.3)	22%	10,528.7 (8,600.5–12,716.4)	12,917.2 (10,630.6–15,432.4)	23%
Schizophrenia	771.1 (443.1–1,144.3)	1,052.2 (583.9–1,591.4)	36%	781.6 (453.6–1,153.9)	1,074.7 (606.8–1,614.1)	37%
Alcohol use disorders	691 (381.2–1156.4)	757.1 (401.2–1,235.8)	10%	797 (482.4–1,257.7)	882.1 (514.8–1,385.4)	11%
Drug use disorders	1,644.7 (1,176.5–2,156.1)	1,784.5 (1,277.6–2,334.5)	9%	1,873.5 (1,391–2,416.5)	2,076 (1,536.1–2,677.5)	11%
Opioid use disorders	746.5 (502.9–1003.1)	844.4 (571.9–1,134.8)	13%	886.9 (629.1–1,165.1)	1,016.2 (728.8–1,339.5)	15%
Cocaine use disorders	195.6 (112.9–306.5)	216.7 (124.3–335)	11%	199.3 (117.2–313.6)	218.6 (124.8–338.9)	10%
Amphetamine use disorders	155.2 (86.9–249.2)	162.1 (91.4–255.5)	4%	157.2 (88.8–251.8)	164.5 (92.3–262.9)	5%
Cannabis use disorders	213.6 (107.1–377.3)	201.5 (100.3–367.1)	−6%	213.6 (107.1–377.3)	201.5 (100.3–367.1)	−6%
Other drug use disorders	333.8 (205.6–499.4)	359.8 (226.7–523.5)	8%	416.4 (280.9–592.2)	475.1 (327–669.2)	14%
Unipolar depressive disorders	4,011.1 (2,829.7–5,395.6)	5,308.1 (3,910.9–7,025.8)	32%	4,011.1 (2,829.7–5,395.6)	5,308.1 (3,910.9–7,025.8)	32%
Major depressive disorder	3,352.4 (2,332.1–4,626.6)	4,521.8 (3,227.7–6,093.6)	35%	3,352.4 (2,332.1–4,626.6)	4,521.8 (3,227.7–6,093.6)	35%
Dysthymia	658.7 (426.3–922.7)	786.3 (508.1–1,094.9)	19%	658.7 (426.3–922.7)	786.3 (508.1–1,094.9)	19%
Bipolar affective disorder	696.1 (425.1–1051)	835.3 (510.6–1,251.2)	20%	696.1 (425.1–1051)	835.3 (510.6–1251.2)	20%
Anxiety disorders	1,213.6 (826.9–1,673.9)	1331.1 (906.8–1,856.4)	10%	1,213.6 (826.9–1,673.9)	1,331.1 (906.8–1,856.4)	10%
Eating disorders	296.4 (171.5–476.8)	570.1 (334.2–924.9)	92%	298.7 (174.6–479.3)	574.6 (339.7–929.5)	92%
Pervasive development disorders	399.8 (269.4–578.2)	452.2 (302.8–648.9)	13%	399.8 (269.4–578.2)	452.2 (302.8–648.9)	13%
Autism	188.8 (122.7–275.7)	216.3 (140.4–317.6)	15%	188.8 (122.7–275.7)	216.3 (140.4–317.6)	15%
Asperger’s syndrome	211.1 (134.8–319.5)	235.8 (154.4–352.7)	12%	211.1 (134.8–319.5)	235.8 (154.4–352.7)	12%
Childhood behavioral disorders	291.8 (171.3–450.9)	209.7 (120.2–323.4)	−28%	291.8 (171.3–450.9)	209.7 (120.2–323.4)	−28%
Attention-deficit hyperactivity disorder	27 (15.2–43.6)	20.8 (11.8–33.8)	−23%	27 (15.2–43.6)	20.8 (11.8–33.8)	−23%
Conduct disorder	264.7 (151–419.6)	188.9 (105–299.2)	−29%	264.7 (151–419.6)	188.9 (105–299.2)	−29%
Idiopathic intellectual disability	92 (53.5–142.5)	74.1 (38–123.4)	−19%	92 (53.5–142.5)	74.1 (38–123.4)	−19%
Other mental and behavioral disorders	69.5 (41–108.2)	89.7 (52–143.5)	29%	73.4 (44.8–112.2)	99.2 (60.1–152.2)	35%
Diabetes, urogenital, blood, and endocrine diseases	4,245.7 (3,050.2–5,693.7)	5,381.9 (3,956.8–7,145.9)	27%	7,013.4 (5,814.5–8,532.3)	8,414.8 (6,968.8–1,0395.6)	20%
Diabetes mellitus	2,207.8 (1461.1–3173)	2,807.9 (1,906.5–4,022.5)	27%	3,490.7 (2,731.4–4,487.2)	3,954.2 (3,014.9–5,187.7)	13%
Acute glomerulonephritis	0 (0–0.1)	0 (0–0)	−9%	3.8 (2.4–6.7)	2.5 (1.1–3.9)	−34%
Chronic kidney diseases	537.1 (370.6–805.8)	770.3 (538.4–1101)	43%	1,371.7 (1,146.5–1,649.9)	1,590.2 (1,313.1–1,967.6)	16%
Chronic kidney disease due to diabetes mellitus	80.8 (52.9–117.2)	119.3 (78.1–172.1)	48%	214.8 (173.7–261.2)	242 (190.6–308.7)	13%
Chronic kidney disease due to hypertension	99.2 (66.2–147.1)	143.9 (97.4–204.3)	45%	264.7 (219.2–322.4)	315.3 (257.2–393.6)	19%
Chronic kidney disease unspecified	357.1 (240.4–547.6)	507.1 (352.2–735)	42%	892.2 (740.5–1101.4)	1,032.9 (841.5–1,303.6)	16%
Urinary diseases and male infertility	395.5 (254.4–603.1)	541.2 (347.1–812.7)	37%	604.2 (451.8–809.5)	963.5 (717.5–1,265.4)	59%
Tubulointerstitial nephritis, pyelonephritis, and urinary tract infections	5.9 (2.6–11.2)	7.4 (3.5–14.3)	27%	156.5 (124.4–229.1)	328.8 (177.1–474.9)	110%
Urolithiasis	50 (27.3–81.3)	72.6 (40.1–114.7)	45%	59.1 (36–91.8)	78.8 (46.4–122.1)	33%
Benign prostatic hyperplasia	319.6 (201.5–487.1)	431.8 (270.1–654.1)	35%	319.6 (201.5–487.1)	431.8 (270.1–654.1)	35%
Male infertility	3.2 (1.2–7.1)	4.2 (1.6–9)	32%	3.2 (1.2–7.1)	4.2 (1.6–9)	32%
Other urinary diseases	16.9 (9.5–27.4)	25.1 (14.1–40.3)	49%	65.9 (51.6–86)	119.8 (87.9–155.5)	82%
Gynecological diseases	464.2 (258.8–797)	585.2 (325.2–999)	26%	468.9 (263.7–801.4)	590.4 (330–1,004)	26%
Uterine fibroids	96.8 (43.5–190.7)	139.2 (60.2–268.9)	44%	97.2 (43.9–191.1)	139.7 (60.8–269.4)	44%
Polycystic ovarian syndrome	142.4 (66.4–265.2)	170.3 (77.9–324.2)	20%	142.4 (66.4–265.2)	170.3 (77.9–324.2)	20%
Female infertility	2.5 (0.9–5.4)	3.1 (1.1–6.7)	24%	2.5 (0.9–5.4)	3.1 (1.1–6.7)	24%
Endometriosis	27.1 (9–52.7)	34.3 (12.4–66.5)	27%	27.1 (9–52.7)	34.3 (12.4–66.5)	27%
Genital prolapse	123.8 (49.5–259.5)	156.3 (62.6–339.3)	26%	124.2 (49.9–259.9)	156.7 (63–339.7)	26%
Premenstrual syndrome	71.7 (0–200.2)	82.1 (0–213.6)	14%	71.7 (0–200.2)	82.1 (0–213.6)	14%
Other gynecological diseases	0 (0–0)	0 (0–0)	–*	3.9 (3.4–4.6)	4.2 (3.3–5.4)	10%
Hemoglobinopathies and hemolytic anemia	498.7 (296.2–1080.4)	477.4 (291.8–935)	−4%	599.4 (393.7–1,169.3)	540.7 (350.5–997.4)	−10%
Thalassemia	161.4 (102.1–247.1)	156.1 (99.3–254.6)	−3%	174.9 (115.5–260.7)	162.3 (105.8–260.9)	−7%
Sickle cell disorders	309.9 (171.6–832.4)	296.2 (172.1–703.8)	−4%	338.4 (199.5–859.6)	309.3 (184.8–715.3)	−9%
G6PD deficiency	24.8 (11.7–93.6)	18.3 (9.3–58.9)	−26%	32.5 (19–101.1)	22.5 (13.2–62.6)	−31%
Other hemoglobinopathies and hemolytic anemia	2.5 (0.5–1.8)	6.7 (0.9–9.3)	169%	53.6 (36.2–64.7)	46.6 (31.4–79.2)	−13%
Other endocrine, nutritional, blood, and immune disorders	142.4 (83.1–228.4)	199.8 (115.9–362)	40%	474.8 (329.2–710.6)	773.4 (529.7–1,539.3)	63%
Musculoskeletal disorders	11,220.4 (,8585.2–14,277.6)	14,278.5 (10,905.8–17,775.5)	27%	11,469.4 (88,29.8–14,558.2)	14,654.1 (11,291.8–18,160.2)	28%
Rheumatoid arthritis	477.5 (345.2–636.5)	665.5 (465.5–881)	39%	519.4 (384.9–684.3)	710.1 (509.5–925.6)	37%
Osteoarthritis	1,055.3 (648.4–1,604.3)	1,407.1 (877.8–2,066.2)	33%	1,055.3 (648.4–1,604.3)	1,407.1 (877.8–2,066.2)	33%
Low back and neck pain	7,363.4 (5,148.2–9,886.3)	9,178.1 (6,357.5–12,295.1)	25%	7,363.4 (5,148.2–9,886.3)	9,178.1 (6,357.5–1,2295.1)	25%
Low back pain	4,784.5 (3,305.8–6,484.6)	6,011.7 (4,104.5–8,080.9)	26%	4,784.5 (3,305.8–6,484.6)	6,011.7 (4,104.5–8,080.9)	26%
Neck pain	2,578.9 (1,769.5–3,608.8)	3,166.3 (2,193.9–4,438)	23%	2,578.9 (1,769.5–3,608.8)	3,166.3 (2,193.9–4,438)	23%
Gout	21.6 (13.4–32.1)	30.2 (19–45.1)	40%	21.6 (13.4–32.1)	30.2 (19–45.1)	40%
Other musculoskeletal disorders	2,302.7 (1,585.5–3,228.6)	2,997.7 (2,089.4–4,136.6)	30%	2,509.7 (1,803.7–3,433.4)	3,328.7 (2,440.8–4,471.4)	33%
Other non-communicable diseases	4,700.2 (3,172.4–6,899.1)	5,087.8 (3,400.3–7,616.6)	8%	6,009.2 (4,467.8–8,218.2)	5,881.5 (4,184.3–8,375.2)	−2%
Congenital anomalies	130.9 (93.7–182.5)	141 (101.2–201.8)	8%	1,278.3 (1,065.6–1,416.6)	778.7 (685.8–973.5)	−39%
Neural tube defects	11.6 (6.4–18.7)	8.2 (4.4–13.6)	−29%	99.4 (58.5–126.5)	18.5 (12.5–28)	−81%
Congenital heart anomalies	34.2 (17.6–66.8)	32.4 (15.9–67.4)	−5%	566.6 (460.8–650.2)	321.4 (254.9–411.9)	−43%
Cleft lip and cleft palate	5.4 (2.7–9.7)	6 (3.1–10.5)	12%	7.9 (4.9–12.4)	7.4 (4.3–12.1)	−6%
Down’s syndrome	38.1 (21.6–61.4)	46.5 (27.1–74.6)	22%	104 (69.3–138.5)	104.7 (80.1–136)	1%
Other chromosomal abnormalities	14.5 (8–23.9)	17.4 (10.1–29.2)	19%	59.4 (41.1–78.5)	55 (39.2–77.1)	−7%
Other congenital anomalies	27.1 (19.1–38.3)	30.6 (21.8–44.9)	13%	441 (294.4–539.7)	271.6 (215.5–397.8)	−38%
Skin and subcutaneous diseases	1,989.9 (1,235.6–3,086)	2,206.8 (1,347–3,476.9)	11%	2,058.2 (1,302–3,150.8)	2,301.8 (1,445–3,558.5)	12%
Eczema	462.2 (233.8–802)	511.7 (252.5–845.6)	11%	462.2 (233.8–802)	511.7 (252.5–845.6)	11%
Psoriasis	87.3 (42.4–145.8)	105.1 (50–172.9)	20%	87.3 (42.4–145.8)	105.1 (50–172.9)	20%
Cellulitis	11.1 (1.2–45.6)	11 (1.3–45.3)	−1%	26.2 (15.7–60.2)	29.6 (16.9–63.8)	13%
Abscess, impetigo, and other bacterial skin diseases	41.8 (16.9–85.1)	49.3 (19.8–101.3)	18%	63.3 (38–107.4)	76 (45.7–129.6)	20%
Scabies	22.9 (10.1–44.9)	26.2 (11.8–49)	15%	22.9 (10.1–44.9)	26.2 (11.8–49)	15%
Fungal skin diseases	107.6 (33.8–246.6)	128.2 (39.3–291.8)	19%	107.6 (33.8–246.6)	128.2 (39.3–291.8)	19%
Viral skin diseases	189.2 (72.5–359.4)	182.5 (66.3–336.4)	−4%	189.2 (72.5–359.4)	182.5 (66.3–336.4)	−4%
Acne vulgaris	312.6 (139.6–589.8)	237.6 (106.6–469.3)	−24%	312.6 (139.6–589.8)	237.6 (106.6–469.3)	−24%
Alopecia areata	78.7 (23–158.4)	91.1 (25.1–182.4)	16%	78.7 (23–158.4)	91.1 (25.1–182.4)	16%
Pruritus	179.4 (82.5–347.4)	244.2 (110.2–489.4)	36%	179.4 (82.5–347.4)	244.2 (110.2–489.4)	36%
Urticaria	137.4 (55.3–236.9)	159 (65–271)	16%	137.4 (55.3–236.9)	159 (65–271)	16%
Decubitus ulcer	30 (9.8–69.2)	40.3 (12.9–96.9)	34%	61.5 (39.8–101.6)	89.7 (55–150.1)	46%
Other skin and subcutaneous diseases	329.6 (155.1–610.6)	420.8 (193.8–806.2)	28%	329.8 (155.2–610.6)	421 (193.9–806.3)	28%
Sense organ diseases	1637 (1164–2335)	1,850.2 (1291.1–2,656.9)	13%	1,637 (1,164–2,335)	1,850.2 (1,291.1–2,656.9)	13%
Glaucoma	36 (23–54.9)	56.9 (37.6–82.7)	58%	36 (23–54.9)	56.9 (37.6–82.7)	58%
Cataracts	193.1 (136.8–261.7)	170.7 (116.3–233.8)	−12%	193.1 (136.8–261.7)	170.7 (116.3–233.8)	−12%
Macular degeneration	58.4 (37.5–87.3)	107.8 (71.8–152.9)	85%	58.4 (37.5–87.3)	107.8 (71.8–152.9)	85%
Refraction and accommodation disorders	84 (61.3–110.7)	107.9 (77.6–142.3)	28%	84 (61.3–110.7)	107.9 (77.6–142.3)	28%
Other hearing loss	926.8 (541.4–1480.3)	962.7 (554.5–1598.5)	4%	926.8 (541.4–1,480.3)	962.7 (554.5–1,598.5)	4%
Other vision loss	329.9 (174.9–586)	433.2 (222–771)	31%	329.9 (174.9–586)	433.2 (222–771)	31%
Other sense organ diseases	8.8 (3–19.8)	11 (3.7–25.4)	25%	8.8 (3–19.8)	11 (3.7–25.4)	25%
Oral disorders	942.4 (564.3–1507.7)	889.8 (519.4–1403.9)	−6%	942.4 (564.3–1,507.7)	889.8 (519.4–1,403.9)	−6%
Dental caries	110.2 (45–216.1)	130.4 (52.8–250.1)	18%	110.2 (45–216.1)	130.4 (52.8–250.1)	18%
Periodontal disease	143 (54.8–312.3)	193.8 (72.6–400.1)	36%	143 (54.8–312.3)	193.8 (72.6–400.1)	36%
Edentulism	689.2 (394.8–1103.5)	565.6 (322–903)	−18%	689.2 (394.8–1103.5)	565.6 (322–903)	−18%
Sudden infant death syndrome	0 (0–0)	0 (0–0)	–*	93.3 (40.6–150.8)	61 (31.4–111.4)	−35%
**Injuries**	3,873.3 (2,679.9–5,500.9)	5,495 (3,846.6–7,727.1)	42%	11,961.1 (10,559.1–13,749.9)	9,962.5 (8,154–12,170.7)	−17%
Transport injuries	1,134 (763.2–1,670.3)	1,428.6 (975–2,074.9)	26%	4,925.5 (4,315.5–5,683.5)	3,047.6 (2,494.2–3,809.3)	−38%
Road injury	982.9 (659–1,450.2)	1,197.1 (810.2–1,745.7)	22%	4,671.5 (4,049.7–5,342.3)	2,692.9 (2,226.1–3,328.1)	−42%
Pedestrian injury by road vehicle	169 (113.6–248.3)	243.2 (165–350.8)	44%	580.3 (487.4–761.5)	464.4 (372.3–584.4)	−20%
Pedal cycle vehicle	18.8 (12.7–27.8)	50.5 (34.2–74.1)	169%	71.2 (53.8–110.9)	107.2 (75.3–136.5)	51%
Motorized vehicle with two wheels	232.4 (155.6–347.3)	172.6 (116.1–252.7)	−26%	1,423.3 (1,009.6–1,638.8)	436 (360.3–539.5)	−69%
Motorized vehicle with three or more wheels	576.6 (386.6–852.7)	779.7 (527.3–1134.8)	35%	2,579.1 (2,156.9–3,157.2)	1,722.1 (1,368.2–2,279.8)	−33%
Road injury other	13 (8.8–19.2)	12.1 (8.2–17.4)	−7%	44.6 (31.7–60)	24 (17.7–33.7)	−46%
Other transport injury	151.1 (102.4–223)	231.5 (157.9–331.2)	53%	254 (189.8–359.6)	354.8 (275.4–460.4)	40%
Unintentional injuries other than transport injuries	2,638 (1,821–3,727.8)	3,944 (2,760.5–5,515.8)	50%	5,486.5 (4,503.8–6,659)	5,472.7 (4,205.3–7,082.7)	0%
Falls	2,150.2 (1,471.2–3043.3)	3,315.8 (2,316.6–4,644.3)	54%	2,462.8 (1,754.8–3,385.5)	3,763.4 (2,726.7–5,125)	53%
Drowning	9.7 (6.1–14.5)	11.1 (7–16.8)	14%	404.4 (312.7–497.3)	222.8 (181–295.5)	−45%
Fire, heat and hot substances	57 (30.5–106.3)	70.2 (37.6–127.1)	23%	158.1 (122.4–213)	141 (105.1–202.2)	−11%
Poisonings	9.2 (5.6–14.6)	7.1 (4.3–11.3)	−23%	220.6 (153.6–338.5)	165.9 (79.7–233.9)	−25%
Exposure to mechanical forces	72.6 (45.5–114)	75.2 (47.8–113.9)	4%	269.1 (212.6–366.4)	185.4 (130.8–252.4)	−31%
Mechanical forces (firearm)	26.9 (17–42.2)	14.6 (9.4–22.2)	−46%	94.8 (56.5–123)	31.6 (22.7–47.7)	−67%
Mechanical forces (other)	56.5 (35.4–88.2)	79.1 (50.2–119.5)	40%	185.1 (147.6–282.1)	172.3 (131.7–241.7)	−7%
Adverse effects of medical treatment	45.3 (29.2–68.3)	102.6 (66–151.3)	127%	143.5 (115.6–181.7)	227.3 (174.5–307.9)	58%
Animal contact	21.9 (14.2–32.8)	11.7 (7.7–17.2)	−47%	35.3 (26.2–47.7)	18.6 (14–25.1)	−47%
Animal contact (venomous)	14.3 (8.7–22.3)	6.8 (4.3–10.5)	−53%	16.4 (10.6–24.8)	8.3 (5.6–12.2)	−50%
Animal contact (non-venomous)	7.6 (4.8–11.8)	4.9 (3–7.3)	−36%	18.9 (13.9–24.7)	10.3 (7.8–13.9)	−45%
Unintentional injuries not classified elsewhere	272.1 (185.1–396.4)	350.3 (232.7–505.7)	29%	1,792.7 (1,054.8–2,031)	748.2 (605.7–955.4)	−58%
Self-harm and interpersonal violence	101.3 (66.6–146.4)	122.3 (81.1–173)	21%	1,549.1 (1,277.2–2,016.4)	1,442.2 (1,051.7–1,690.9)	−7%
Self-harm	12.6 (7.9–18.3)	15.2 (9.7–23.1)	21%	1,244.6 (9,62.4–1,686.8)	1,154.4 (7,59.7–1,377.5)	−7%
Interpersonal violence	88.7 (58.4–129.4)	107.1 (70.8–151.4)	21%	304.5 (241.9–403)	287.8 (230.5–369.8)	−5%
Assault by firearm	23.2 (15–34.3)	30.1 (19.5–42.9)	30%	74.6 (59–105.3)	74.2 (57.3–98.9)	−1%
Assault by sharp object	31.9 (20.9–46.9)	56.2 (37–79.6)	76%	106.7 (79.1–175.7)	143.2 (94.8–186.6)	34%
Assault by other means	40.1 (26.5–57.7)	31.3 (21–43.8)	−22%	129.7 (98.4–158.1)	80.9 (65.5–106)	−38%

Data are presented as mean values and uncertainty intervals (Lower, Upper).

*– not applicable.

Putting premature mortality and disability together in terms of DALYs provides an overall picture of the leading health problems in Spain. Between 1990 and 2010, there was a slight increase (5.4%) in DALYs (i.e., overall health burden) in the overall Spanish population (from 104,615.9 in 1990 to 110,269.4 in 2010). The dominant causes of DALYs in 2010 were neoplasms, cardiovascular and circulatory diseases, musculoskeletal disorders, mental and behavioral disorders, and diabetes, urogenital, blood, and endocrine diseases, which accounted for nearly 59% of all DALYs (Table 3).

**Figure 3:**
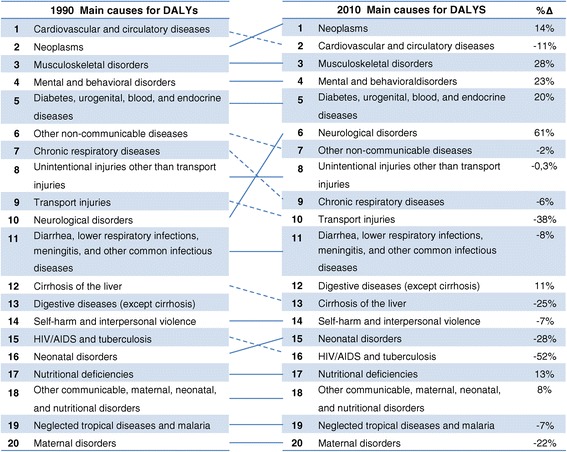
**Spanish disability-adjusted life years (DALYs) ranks for the top 20 main causes in 1990 and 2010, and the percentage change between 1990 and 2010.** Continuous line represents an ascending order in rank and the broken line represents a descending order. (Δ%) = % of change in absolute numbers of DALYs.

The top five leading causes for DALYs remained similar in both 1990 and 2010, despite the decreases or increases in the number of DALYs for each cause (% of change between the numbers of DALYs). There was only a slight change in numerical order between the first two causes. Due to a large decrease in the number of DALYs (11%), cardiovascular and circulatory diseases dropped to the second cause of DALYs in 2010 and neoplasms reached the first position increasing by 14% from 1990. Between the sixth and the eleventh positions the causes for DALYs changed only slightly. The transport injuries dropped from the ninth position in 1990s to the tenth in 2010, due to a large decrease (38%) in the number of DALYs. The neurological disorders, with a 61% increase in DALYs, achieved the sixth position in 2010 from its tenth position in 1990. The HIV/AIDS and tuberculosis cause dropped one position and neonatal disorders increased one position in the list of causes for DALYs for the year 2010. Finally, the last four causes among the 20 top ranking of DALYs in Spain remained completely stable between 1990 and 2010, despite the changes in disability (Figure 3).

**Figure 4:**
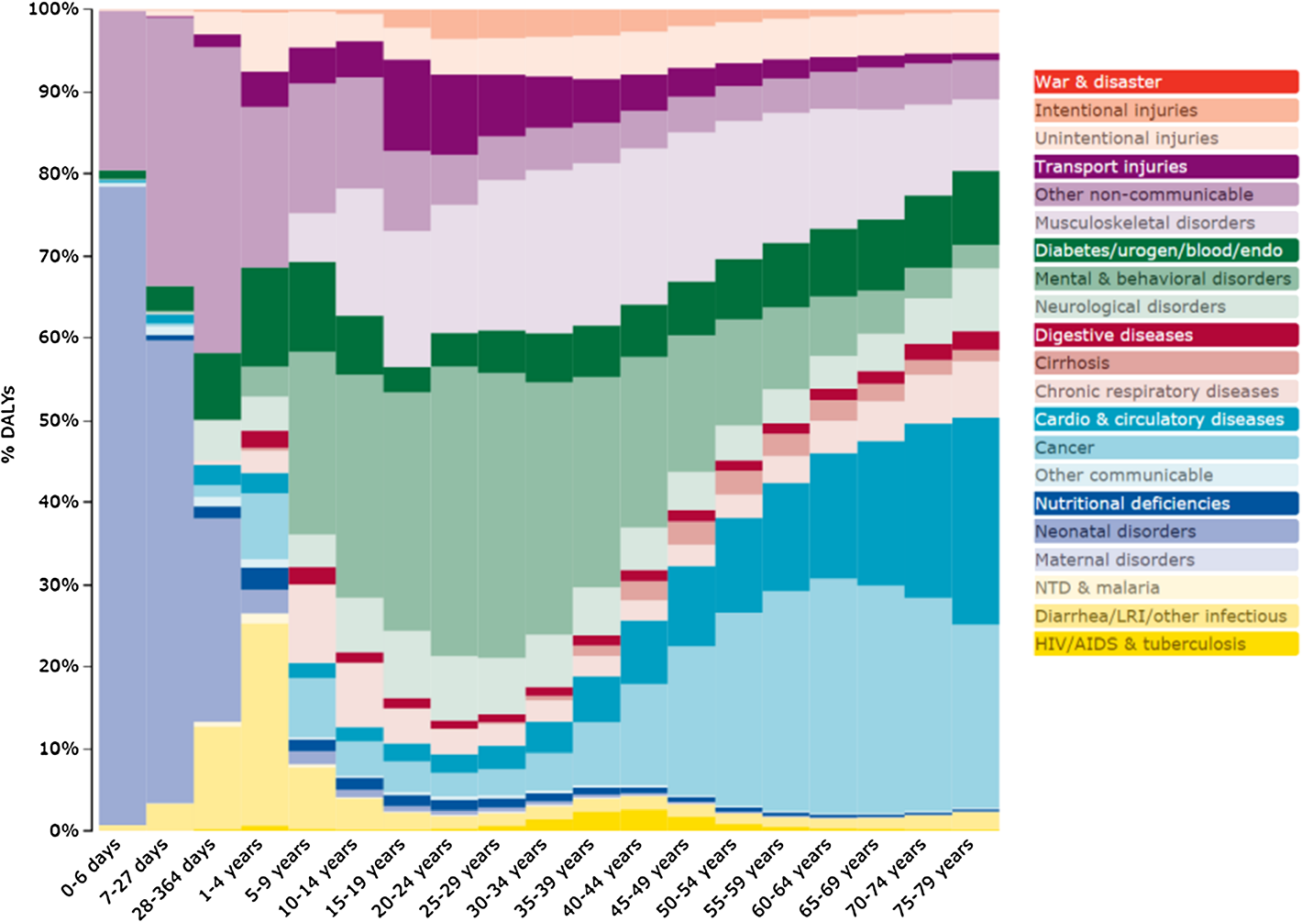
**DALYs in Spain by cause and age in 2010.** Adopted and modified from GBD data visualizations.

Summarizing the above data, in 2010, the leading causes for DALYs among newborn children (0–1 years old) were neonatal disorders and the other communicable diseases group. The leading causes for DALYs in the younger group (5 to 44 years old) in the Spanish population were mental and behavioral disorders and musculoskeletal disorders, while in the middle aged and older adults groups the leading causes shifted to cardiovascular and circulatory diseases and neoplasms (Figure 4).

**Figure 5:**
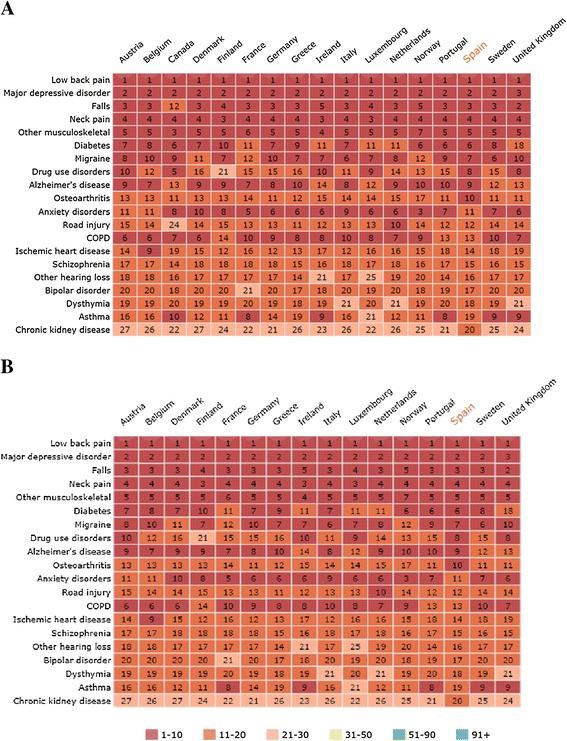
**Age-standardized YLDs ranking relative to comparator European countries by the top 20 causes in (A) 1990 and (B) 2010.** Numbers in cells indicate the ranks by country for each cause, with 1 being the disorder with the highest impact. The presented causes are ordered by the 20 leading causes of YLDs in Spain. YLDs, Years lived with disability; COPD, Chronic obstructive pulmonary disease. Adopted and modified from GBD data visualizations.

In both 1990 and 2010, there was a high consistency across European countries regarding the top causes of YLDs being major depressive disorders, musculoskeletal disorders, low back and neck pain, and diabetes, as well as injuries (i.e., falls). YLDs caused by asthma, anxiety disorders, and chronic obstructive pulmonary disease ranked lower in Spain compared with other southern European countries, in both 1990 and 2010 (Figure 5).

From 1990 to 2010 overall DALYs attributable to non-communicable diseases in Spain increased by 897,900 from 8,626,000 to 9,523,900. When standardizing the DALYs of 2010 to the 1990 population, the number of DALYs which would have increased was estimated to be 1,250,900. Thus, the actual population health improved since the demographic changes would have caused a higher increase in DALYs than the actual 2010 DALYs. Changes in DALYs mostly correspond to population increase and are secondary to population ageing. Moreover, the highest difference was attributed to those 40 years and older.

## Discussion

During the past 20 years, substantial changes have taken place in the relative impact of burden of diseases in Spain: while musculoskeletal disorders have increased by 28% (rank 3 as major cause of DALYs), mental and behavioral disorders have increased by 23% (rank 4), neurological disorders by 61% (rank 6), and neoplasms by 14% (rank 1). On the other hand, cardiovascular and circulatory diseases have decreased by 11% (now rank 2), transport injuries by 38% (rank 10), and chronic respiratory diseases by 6% (rank 9). Despite the population growth of 15% between 1990 and 2010 and the ageing of the population, the burden of disease increased only by 5%. The analysis of changes by age and gender group denoted that DALYs decreased when analyzed as rates per 100,000 population.

The relevance of neoplasms and cardiovascular and circulatory diseases on population health is mostly driven by mortality. Accordingly, the cardiovascular and circulatory diseases, neoplasms, and injuries due to transport reasons were the top three leading causes for the burden of YLLs. However, the impact of musculoskeletal disorders and mental disorders is mostly through YLDs, since they are the first and second causes in the ranking. Together, these two groups of disorders, accounted for almost the half (48%) of all YLDs in Spain. Additionally, depression, and other mental disorders (like anxiety, etc.) have been associated to various musculoskeletal disorders [17,18]. All these conditions are related to occupational risk, particularly low back pain [19], and absenteeism. The impact of these disorders and the need for mental health promotion and musculoskeletal health prevention may have been underestimated by public health authorities and policies [20].

Other studies have previously evaluated the burden of morbidity and mortality in Spain [8,9]. According to these, in 2008, the major causes for mortality in males and females were also cardiovascular diseases and malignant neoplasms [8]. Furthermore, a recent study in Valencia reported similar results in mortality rates in the local population (i.e., 26% of all deaths due to malignant tumors and 34% due to cardiovascular diseases [9]).

Clear gender differences emerged in the analyses. Specifically, in males, cancer (i.e., neoplasms) was the main mortality cause followed by cardiovascular diseases, while in females the order was reversed. Risk factor differences may be causing the increased impact of cancer in males: they still have higher rates of smoking and heavy alcohol consumption [21,22]. The increased mortality of cardiovascular diseases in females is due to cerebro-vascular problems. Further, hormonal factors (disappearance of the protective role of estrogens after menopause) have been associated to the increased risk of stroke in females [23].

The top five causes for the burden of YLDs in Spain are similar to those in the other Mediterranean countries (except for slight differences with France) [24-26]. Similar results appeared also in the burden of DALYs comparing Spain to other European countries [24-26]. These similarities in the Mediterranean population’s health could be a result of the common lifestyle habits (dietary habits, smoking, physical activity, etc.) [27], as well as shared genetic traits within the region [28]. Spain, as well as other Mediterranean countries, has to shift the provided health care services from curative to preventive [29,30] and to identify the priority diseases for health research funding and prevention policies development [8].

When comparing the results among all European countries, some remarkable results have also been found. While there are substantial differences in the rank order of diseases regarding DALY’s [7], there are much less differences in the first five causes of YLDs across European countries. Country differences may be mostly caused by differences in mortality instead of differences in YLDs. Whether this can be attributed to mortality figures being recorded more systematically in each country than prevalence figures should still be clarified.

In 2010, Spain presented the same leading conditions for YLLs compared to other Mediterranean countries, such as Greece, France, and Italy [24-26]. In 2010, Spain, France, and Italy showed age-standardized YLL rates for liver cancers significantly higher than the overall mean rate. Spain and Greece showed significantly higher rates of age-standardized YLL rates for bladder cancers [7]. Besides, the leading causes for premature mortality in the UK seem to be similar with those in Spain (i.e., cardiovascular and circulatory diseases and trachea, bronchus, and lung cancers [24-26]).

Globally, there is continuous shift from communicable to non-communicable diseases as the leading causes of mortality and DALYs [2,5]. According to the latest data for 2010, in the top ten leading causes of population’s mortality, five were classified as non-communicable [2]. Global DALYs remained stable from 1990 to 2010 [5]. However, the global age-standardized DALY rates [5] actually decreased, which is consistent with our findings in Spain. Ischemic heart disease was the leading cause of DALYs worldwide in 2010, followed by lower respiratory infections, stroke, diarrheal diseases, and HIV/AIDS. Depressive disorders, also climbed from the fifteenth to the eleventh rank and road injury from twelfth to tenth rank [2,5]. However, in Spain the major causes for DALYs were neoplasms followed by cardiovascular diseases, musculoskeletal disorders, and mental disorders. Neurological disorders moved from the tenth to sixth rank; this high ranking in musculoskeletal disorders was quite similar with the global estimates [5] – low back pain and neck pain are the most important contributors accompanied by osteoarthritis, rheumatoid arthritis, and gout [5,31]. The pathophysiology of chronic diseases, especially cardiovascular diseases and cancers, is complex, with the interaction of new environmental risk factors (e.g., multimorbidity, financial status, etc.), in addition to the classical modifiable risk factors (i.e., cognition reserve, smoking, unhealthy diet, physical activity, hypertension, etc.), making the prevention of chronic diseases in older adults quite difficult. Efforts to improve and protect health, prevent disease and injury, and deliver high-quality health care to the population must be tailored to address the causes associated with the greatest burden mainly of chronic diseases [32]. It is expected that preventive strategies can influence many of the aforementioned chronic conditions and non-communicable causes and increase the quality of life, while averting or minimizing the need for expensive medical care [33,34]. Furthermore, the engagement with the GBD collaborative group will provide more and better analysis data of the global burden of disease and, specifically, in Spain – a fact that will contribute to continuous improvements of the health estimates in future iterations of the GBD study.

### Limitations

The previous results should be considered taking into account the following limitations. Although Spain follows the European Statistics Code of Practice of 2006–2008 and data is collected in a consistent way across the country, there are a number of issues that should be considered when analyzing the results. Regarding mortality, the GBD project defined a number of garbage codes, which are causes of death that should not be identified as underlying causes of death but have been entered as the underlying causes of death on death certificates. Garbage codes were substituted by underlying causes based on pathophysiology. The fraction of garbage codes in Spain was within the world average. The substitution of garbage codes has impact on mortality causes. For example, in 2010, transport injury deaths based on raw data from Spain were 2,257. After correction for garbage codes and other adjustments, this number increased to 3,657 deaths. Following the modeling process, it further increased to 3,950.

In this study, the calculation of DWs were based on surveys including several countries, and not just Spain. However, according to recent results [10], the DW assessments appeared to be consistent even among different cultural environments. Another aspect is the prevalence of the referred health conditions, which was based on epidemiological studies, a fact that may create uncertainty depending on the quality of the primary data. To overcome this issue, some expert groups developed tools to assess the risk of bias in the selected studies and sensitivity analyses were performed to weight study sizes according to their quality, or even to eliminate them from the final analysis [10]. As referred in previous works of the GBD study [10], Bayesian statistical models were used to estimate prevalence of conditions in each country by age, sex, and year. The nature of this estimation process implies that, in some cases, depending on the covariates, estimated variance might be smaller than the real variance across countries in a region, and in some other cases, uncertainty intervals for a specific estimate might be exaggerated. Furthermore, the calculation of uncertainty intervals throughout the Bayesian model analysis has provided some information on the extent of available information for Spain. However, the nature of the estimation process for causes of death and the prevalence of sequelae more generally lead to exaggerated uncertainty intervals in a high-income country such as Spain [12]. These wide uncertainty intervals may limit the number of the detected significant changes in the burden of disease between 1990 and 2010.

## Conclusions

The present findings, together with previous work [5], suggest that cardiovascular and circulatory diseases, neoplasms, and mental and neurological disorders seem to be the leading causes of mortality as well as for YLDs and DALYs in Spain. Although several health promotion action plans have taken place globally [35,36] in the past few years, morbidity throughout non-communicable diseases is increasing at alarming rates. Public health care systems have to focus further on the quality of health care services [37]. Furthermore, health promotion strategies should focus on health education programs that could improve quality of life. Public health care services have to shift from being curative to being preventive; in Spain, a strong emphasis should be given on health promotion, disease prevention, and rehabilitation.

## Abbreviations

DALYs: Disability-adjusted life years
DW: Disability weight
GBD: Global Burden of Diseases, Injuries, and Risk Factors
UK: United Kingdom
YLDs: years lived with disability
YLLs: Years of life lost
